# *Lycium barbarum* (Goji berry) mouthwash is a viable alternative to 0.2% chlorhexidine gluconate for managing chronic periodontitis: a randomized clinical trial

**DOI:** 10.12688/f1000research.129891.1

**Published:** 2023-03-20

**Authors:** Amee Sanghavi, Laasya Shettigar, Aditi Chopra, Ashmeet Shah, Richard Lobo, Padmaja A Shenoy, ShivaPrasada Gadag, Usha Y Nayak, Mangalore Shravya S, Shobha Ullas Kamath, Prajna P Nayak

**Affiliations:** 1 Manipal College of Dental Sciences, Manipal Academy of Higher Education, Manipal, Karnataka, 576104, India; 2 Periodontology, Manipal College of Dental Sciences, Manipal Academy of Higher Education, Manipal, Karnataka, 576104, India; 3 Department of Pharmacognosy, Manipal College of Pharamcuetical Sciences, Manipal Academy of Higher Education, Manipal, Karnataka, 576104, India; 4 Microbiology, Kasturba Medical College and Hospital, Manipal Academy of Higher Education, Manipal, Karnataka, 576104, India; 5 Pharmacuetics, Manipal College of Pharmacuetical Sciences, Manipal Academy of Higher Education, Manipal, Karnataka, 576104, India; 6 Biochemistry, Kasturba Medical College and Hospital, Manipal Academy of Higher Education, Manipal, Karnataka, 576104, India; 7 Public Health Dentistry, Manipal College of Dental Sciences, Manipal Academy of Higher Education, Manipal, Karnataka, 576104, India

**Keywords:** Periodontitis, Periodontal disease, Oral health, Dental Hygiene, Lycium Barbarum, Goji berry, Mouthwash, Chlorhexidine, Herbal, Antioxidants

## Abstract

Background: Removal of the microbial deposits (plaque and calculus) by performing effective scaling and root planing (SRP) is the primary and fundamental requirement for managing periodontal disease. Various adjuncts with antimicrobial, antioxidant, and anti-inflammatory properties are used as adjuncts to SRP for managing chronic periodontitis. However, with a rapid rise in antimicrobial resistance to several antimicrobial agents along with the increased risk of adverse effects, the use the conventional chemotherapeutic agents for managing periodontal disease is slowly declining. Hence, there is a urgent need to explore new plant-based products for treating periodontal disease. *Lycium barbarum (L. barbarum*), or goji berry, has recently gained popularity for managing chronic inflammatory and infectious diseases. However, its efficacy in managing periodontal diseases has never been explored. Hence the present study aims to evaluate the efficacy of *L. barbarum* mouthwash along with SRP compared to chlorhexidine for managing chronic periodontitis.

Methods: The study is designed as a randomized clinical trial with 57 adult participants (Males:29; Females:28) with chronic periodontitis. The participants were divided randomly into two groups: One group used *L. barbarum* mouthwash and the other group used ‘0.2% chlorhexidine gluconate’ mouthwash. The changes in the gingival index (Gi), plaque index (Pi), bleeding on probing (BOP), clinical attachment loss (CAL), probing pocket depth (PPD), microbial load, and antioxidant levels (protein thiol ) in saliva were noted at the baseline, at 15 days and one month.

Results: A statistically significant difference was noted in the Pi (P-value = 0.791), Gi (P-value= 0.594), PPD (with P-value= 0.134), and microbial levels (P-value = 0.188) in both groups from the baseline. The protein thiol levels in saliva were increased only in the goji berry group.

Conclusion: *L. barbarum* mouthwash along with SRP was found to be effective in managing periodontal disease.

## Introduction

Periodontitis is defined as a chronic immuno-inflammatory multifactorial disease that affects the soft tissue around the teeth. ^1^ Periodontitis is considered the 6th most common disease that affects approximately 20–50% of the adult population worldwide. ^2^ It is more prevalent in males as compared to females. It is primarily caused by the interaction of the gingival tissues with the microorganisms in the oral biofilm. This host-microbial interaction triggers a massive influx of various pro-inflammatory mediators, microbial byproducts, proteolytic enzymes, and free reactive oxygen species, in the gingival and periodontal tissues leading to periodontal tissue destruction. ^3^^–^^6^ Due to increased oxidative stress during periodontal disease, it is also referred to as free radical-mediated tissue injury. ^7^ Some of the common putative periodontal microbes associated with chronic periodontitis are *Porphyromonas gingivalis, Tanerella forsythia, Treponema denticola, Campylobacter rectus,* and *Fusobacterium nucleatum.* ^7^ Apart from the microbial etiology, other risk factors that exaggerate periodontal inflammation include smoking, diabetes mellitus, HIV, nutritional deficiency, medications, poor oral hygiene, and genetics. ^8^

The primary and most vital step to control periodontal inflammation is to control the biofilm formed around the teeth by effective mechanical debridement by either hand or machine-driven instruments. ^9^^,^^10^ Effective scaling and root planning (SRP) can reduce gingival inflammation, thereby preventing disease progression and restoring gingival health. ^11^ However, mechanical debridement is technically demanding, as many times removal of hard and soft tissue deposits from pockets greater than five mm becomes challenging. Additionally, it is difficult to completely remove the smooth or burnished calculus from deep and circuitous periodontal pockets, furcation areas, root concavities, and irregular roots owing to a lack of good visibility and accessibility to such areas. ^12^^,^^13^ Hence SRP in deep and tortuous pockets often exhibits residual subgingival biofilm and calculus, thereby warranting a need for additional periodontal therapy. ^12^^–^^15^

The efficacy of professional prophylaxis is also dependent on the individuals’ compliance and motivation to maintain a meticulous oral care regime and effective plaque control at home. ^16^ SRP alone may not be sufficient to maintain the required plaque control if patient compliance is poor and the patient does not effectively follow oral hygiene instructions at home. Studies have shown that even after a good plaque control regime, posterior, palatal, and lingual surfaces of the teeth will retain some amount of plaque, and this warrants the need for adjuncts to regular toothbrushes at home. ^11^^,^^17^^–^^23^

Chemical plaque control measures are often advocated as adjuncts to professional oral prophylaxis and daily home care. ^24^ Various chemical plaque control agents that have antimicrobial and anti-inflammatory are being used in the form of mouthwash, gels, gum paints, fibers, varnishes, microspheres, chips, tablets, powder, and capsules for managing gingivitis and chronic periodontitis. Among all these agents, chlorhexidine gluconate is considered the most popular and routinely used agent for managing periodontal disease. However, prolonged use of chlorhexidine is contraindicated owing to various side effects like altered taste sensation, staining of the teeth and soft tissues such as tongue and mucosa, increased calculus formation, and parotid gland swellings. ^25^ Chlorhexidine has also been shown to have cytotoxic effects on the gingival fibroblasts, ^26^ periodontal ligament, ^27^ and osteoblastic cells. ^28^ Recent studies have even reported the emergence of antimicrobial resistance among oral bacteria to chlorhexidine molecules. ^29^ Thus, there is an emerging trend to use natural and herbal extracts for treating periodontal disease. Herbal extracts from neem, Tulsi, guava, green tea, turmeric, curcumin, pomegranate, and many more plants have been tried to effectively treat gingival and periodontal diseases. ^30^^–^^34^ Recently, goji berry, commonly known as Wolfberry, Himalayan goji, or Tibetan goji, has gained a lot of popularity due to its strong antioxidant and anti-inflammatory properties.

Goji berry is scientifically known as *Lycium barbarum (L. barbarum).* *L. barbarum* is native to southeast Europe, China, and Asia. It comes under the family of Solanaceae. ^35^ The fruit in the form of berries is consumed in both fresh and dried form. *L. barbarum* contains abundant antioxidants such as scopoletin, vitamin C analogs, carotenoids (zeaxanthin and β-carotene), flavonoids, quercetin, etc. These constituents have powerful antioxidant, immuno-modulating and anticancer properties. ^36^^–^^46^ The stems and berries of goji berry plant was effective against many various Gram-negative bacteria and Gram-positive bacteria. Soesanto et al (2021) also showed that ethanolic of *L. barbarum* was effective against caries-causing bacteria ( *Streptococcus. Mutans)* and *P. gingivalis* at 100 μg/mL. ^47^ Previous in-vitro studies have also found that the minimal inhibitory concentration (MIC) of *L. barbarum* was comparable to chlorhexidine, however, its efficacy was less as compared to the antibiotic doxycycline. ^48^^,^^49^ At 50 μg/mL, ethanolic extract of goji berry could inhibit most of the periodontal pathogens. ^48^^,^^49^

However, no clinical study has yet assessed the effectiveness of goji berry mouthwash as an adjunct to SRP for the management of periodontal diseases. Therefore, this clinical study aims to evaluate the efficacy of *L. barbarum* mouthwash along with SRP for patients with chronic periodontitis compared to chlorhexidine for the first time. ^47^

### The objectives of the study include

1. To evaluate the effect of *L. barbarum* mouthwash on the Pi, Gi, BOP, PPD, and CAL at baseline, 15 days and one month compared to 0.2% chlorhexidine gluconate mouthwash.
2. To evaluate and compare the change in the protein thiol levels in saliva at 15 days, and one month compared to baseline.
3. To evaluate and compare the reduction in the microbial count at end of one month compared to baseline in participants using *L. barbarum* mouthwash compared to chlorhexidine mouthwash

## Methods

### Trial design

**Figure 1.**
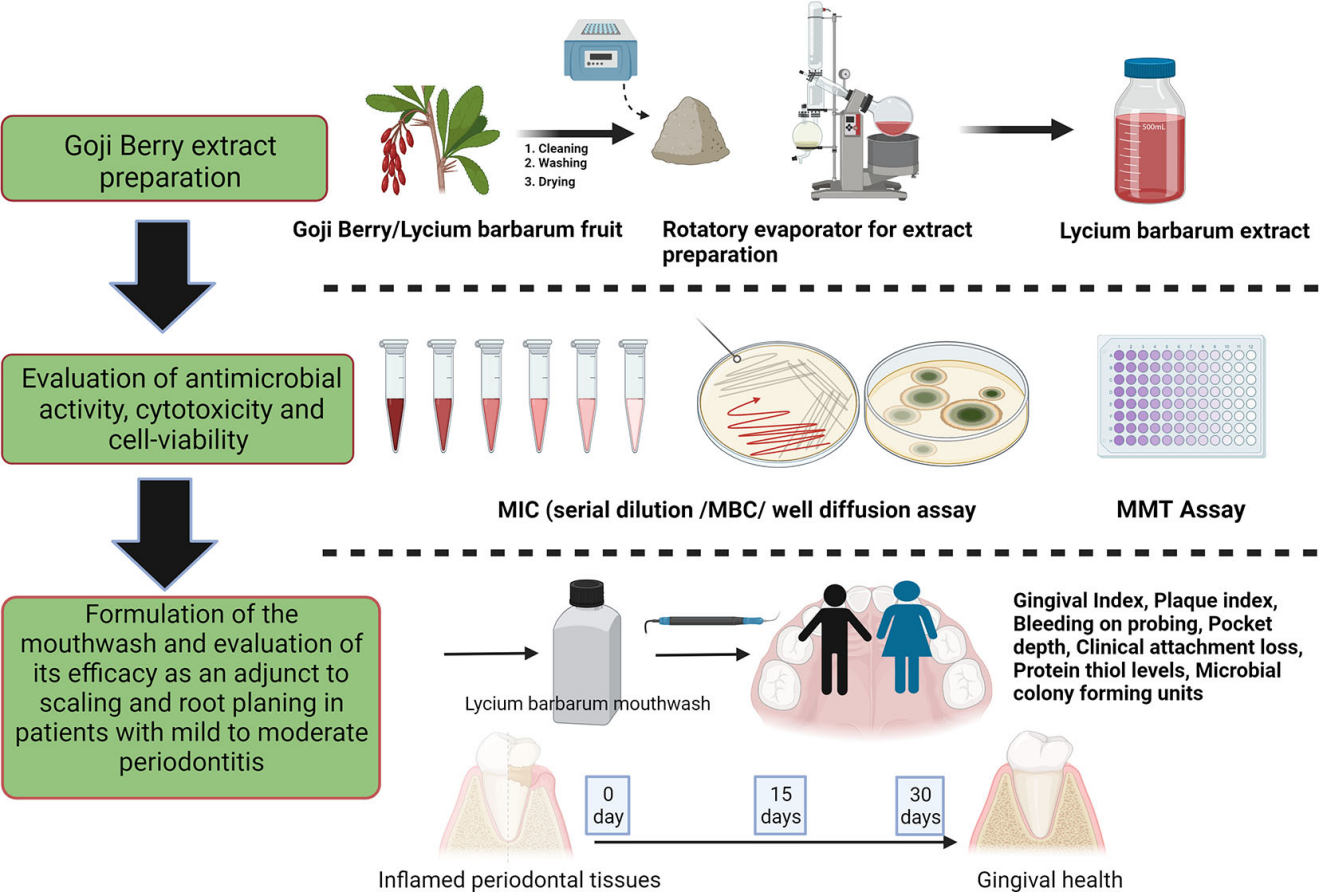
Schematic representation of the study design (Created in Biorender).

**Figure 2.**
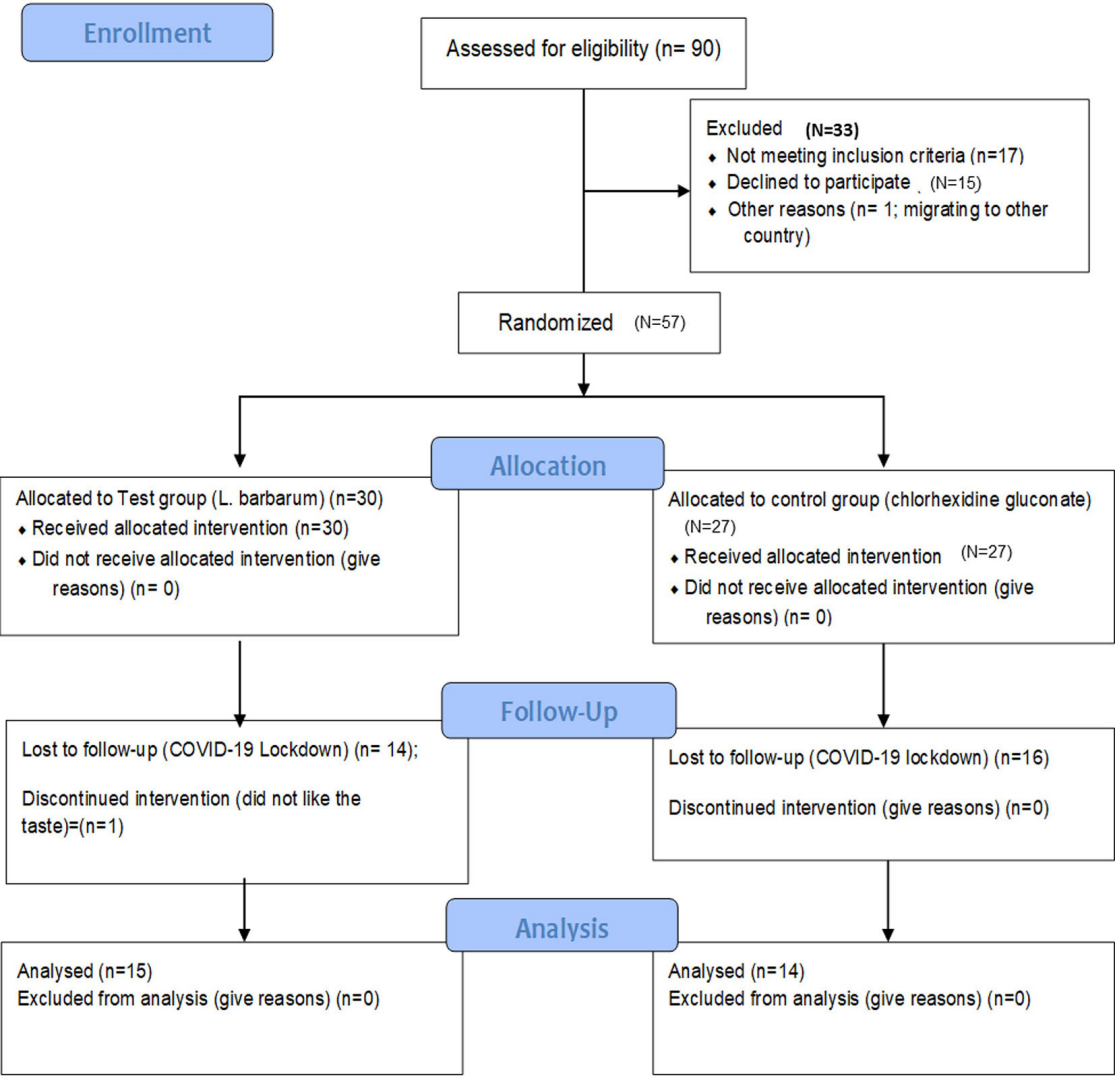
CONSORT flow diagram.

The study was designed as a randomized, double-blind single-centered parallel arm study with an allocation ratio of 1:1. The study was conducted at Manipal’ from 2019 to 2020 following the “Helsinki Declaration of 1975 (as revised in 2000)”. The trial was initiated after receiving ethical clearance from the Kasturba Medical Hospital Institutional Ethics Committee with IEC no 117/2019. The trial has been registered at the ‘Clinical trial registry (CTRI/2019/05/019042)’ and was done following the CONSORT and SAGER guidelines ( Figures 1 and 2). ^48^ The step in the clinical trials are explained as follows:

***Formulation of mouthwash:*** The mouthwash was prepared at the “Manipal College of Pharmaceutical Sciences, Manipal”. *L. barbarum* mouthwash was prepared by preparing an ethanolic extract of dried goji berry using Soxhlet’s method and dissolving the accurately 50 ug/ ml of weighed goji berry extract for formulating the mouthwash as described previously. ^49^ A concentration of 50 ug/ml was mixed in 1.15 of 100% alcohol and then homogenized using of 15% glycerol. To this around 15% propylene glycol, 1% Tween solution was added to formulate the mouthwash. 0.1% menthol was added to the adjust to the taste of the mouthwash. The volume was adjusted to 100% by using distilled water. 100% alcohol was added to serve both as a preservative and a dissolvent.

***Assessment of stability of mouthwash***

The shelf life of the mouthwash was measured as per the ICH guidelines and the samples were tested for three months under three different conditions: “35°±2% C with relative humidity of 60% ± 5%; 25°C± 2°C and relative humidity: 60% ± 5%; and 40°C ± 2°C with a relative humidity of 75% ± 5%”. The mouthwash was kept in opaque plastic bottles and one of the bottles was kept in the stability compartment (Thermo lab, India). The samples were analysed at baseline, one, two, and three months by visual observation and UV spectrum analysis (Shimadzu UV-1601PC, Japan) and analysed for its physical parameters and stability. The mouthwash showed no change in the colour, odour, consistency, or no phase separation at three months. The sample showed no change when observed under the ultraviolet analysis at 274 nm. This indicated that the mouthwash was stable till the end of three months ( Table 1).

**Table 1.** Physical properties and evaluation of the stability of the mouthwash.

Temperature	Evaluation parameters	Observation (in months)			
		Baseline	1	2	3
**Room temperature (3 – 5 ± 2%°C)**	**Visual appearance**	Slight yellowish-brown	Slight yellowish-brown	Slight yellowish-brown	Slight yellowish-brown
	**Precipitation/Phase separation**	Nil	Nil	Nil	Nil
	**Homogeneity**	Good	Good	Good	Good
**Room temperature (25°C ± 2°C) Relative humidity (60% ± 5%)**	**Visual appearance**	Slight yellowish-brown	Slight yellowish-brown	Slight yellowish-brown	Slight yellowish-brown
	**Precipitation/Phase separation**	Nil	Nil	Nil	Nil
	**Homogeneity**	Good	Good	Good	Good
**Room temperature 40°C ± 2°C Relative humidity (75% ± 5%)**	**Visual appearance**	Slight yellowish-brown	Slight yellowish-brown	Slight yellowish-brown	Slight yellowish-brown
	**Precipitation/Phase separation**	Nil	Nil	Nil	Nil
	**Homogeneity**	Good	Good	Good	Good

***Clinical study design***

Around ninety subjects aged 20 to 50 years (both male/females) visiting the outpatient department were screened for the presence of chronic periodontitis according to the following exclusion and inclusion criteria:

***Inclusion criteria**:*

1. Participants in age group of 20-50 years diagnosed with Stage 1 to stage II generalized periodontitis (Grades A to B).
2. Participants with a minimum of 24 functional teeth.
***Exclusion criteria**:*
1. Individuals with severe periodontitis (localized and generalized or Stage III and Stage IV) were excluded.
2. Participants with any allergic reactions to chlorhexidineg
3. Participants with any systemic diseases such as hypertension, diabetes mellitus, cardiovascular, renal, and neurological diseases.
4. Participants on any concurrent medications
5. Participants currently using anti-inflammatory, antibiotics, or analgesics in the last six months
6. Pregnant or lactating mothers
7. Participants with any oral abusive habits such as smoking, alcohol, betel nut chewing, gutka, paan, supari, areca nut
8. Participants who had undergone any periodontal treatment/surgery in the last six months
9. Participants undergoing orthodontic treatment.
10. Participants using any other oral hygiene agents (mouthwash or gels) will be excluded to remove confounding bias arising due to the difference in plaque control measures.

All participants who satisfied the above-mentioned criteria were recruited after obtaining an oral and written signed informed consent.

### Randomization, allocation concealment and blinding

After screening the participants, around 60 participants were recruited after obtaining oral and written informed consent. This sample size was based on p-value <0.05, alpha value = 0.05 two-tailed, power = 0.8, and the effect size = 0.7. Of 60 participants, three participants refused the to take part in the study after scaling, and the remaining fifty-seven participants were randomly allocated using the computer-generated random sequence to either the test group ( *L. barbarum*, n = 30) or the control group (0.2% chlorhexidine gluconate mouthwash, n = 27). The allocation of participants into two groups was done by an investigator who was not the part of the either samples collection, periodontal therapy or periodontal examination. Following recruitment, all participants were assigned to another investigator who was blinded about the randomization and grouping. The participants were also blinded about their group. Following grouping, the following biologic samples were collected:

***a. Microbiology plaque sample collection***

The plaque from the subgingival region was collected from the pocket with the maximum probing depth in each quadrant using a sterile site-specific Gracey curette. After collection, the curette was gently submerged in a reduced transport medium (Thioglycolate bath) for testing the total colony Forming Units (CFU). The number of colonies were calculated on the plated blood agar and then converted to CFU/mL using the following formula: CFU/mL = (no. of colonies × dilution factor)/volume of the culture plate.

***b. Saliva Collection***

Following plaque collection, a stringent method for saliva collection was followed. All participants were requested to sit comfortably in an upright position. Following this, around 2ml of saliva was collected by the ‘spitting method’ and without any stimulation. ^50^ All participants were requested toward spiting the saliva into an Eppendorf vial. The collected saliva was then stored immediately in a refrigerator at -80 degree Celsius. The Eppendorf tubes used to store saliva samples were numerically marked according to the participant number. The saliva collected was sent for biochemical analysis for evaluation of the protein thiol levels using Ellman's Reagent. The absorbance of the agent was measured after incubation at room temperature at 412 nm for around five minutes and the concentration of protein thiol was determined with the standard curve of glutathione.

***c. Examination of the clinical periodontal parameters***

Following saliva sample collection, a periodontal examination was done for all the sextants. The following periodontal parameters were recorded: Gi by Loe & Silness, 1963; percentage of sites with BOP; PI by Silness & Loe 1964; PD and CAL. All the clinical evaluation was done by the investigator who was blinded about the patient’s grouping. The BOP, PPD, and CAL were recorded by Williams periodontal probe (Hu-Friedy, USA). The sites with BOP were checked by noting the presence and absence of bleeding on all four surfaces (buccal, lingual, mesial, and distal) for all the teeth. The percentage of sites with BOP was considered by the percentage of the teeth with BOP to the total teeth present. The PPD and CAL were calculated at buccal, mesial, distal, and lingual. The deepest pocket depth at each surface was recorded. The average of each surface was considered as the reading for that tooth. The mean PPD and CAL were assessed by adding the reading from each tooth and dividing it with the total number of teeth.

Following periodontal examination and sample collection, a thorough SRP was initiated for all the participants. The scaling was assessed by the supervisor to ensure the complete removal of plaque and calculus has been achieved. All patients were educated to brush their/teeth in modified bass technique for two minutes twice daily, to nullify any confounding effects arising due to differences in the oral hygiene measures. All participants were given the opaque amber color bottle which were coded (AX or BX). This was done to blind the patients and investigators regarding the type of mouthwash given to the participants. All patients were instructed to use 10 ml mouthwash diluted with 10 ml of water twice a day for a month. Patients were recalled after 15 days and one month for revaluation. At each recall visit Pi, Gi, % of sites with BOP, CAL, and PPD were noted. The plaque from same site and saliva samples were collected at each recall visit. The investigation for each participant, at each recall visit, was done by the same investigator.

### Statistical analysis

Data obtained was analysed by the ‘SPSS version 26.0, IBM’. The descriptive data such as the frequency and for categorical data; mean and standard deviation for all the numerical data was analysed using ‘Kolmogorov–Smirnov test’. The normality of the distribution was checked for all variables. The inter-group comparisons of all the assessed outcomes were done using an ‘independent sample t-test’. The comparisons between the goji berry mouthwash and chlorhexidine mouthwash was done to measure any significant increase/reduction from baseline to 15 days and one month using Repeated measures ANOVA. Intergroup comparison of reduction of all the variables at follow-up was done using ANCOVA after adjusting the respective baseline scores. The P-value of less than 0.05 was considered to be significant.

## Results

**Figure 3.**
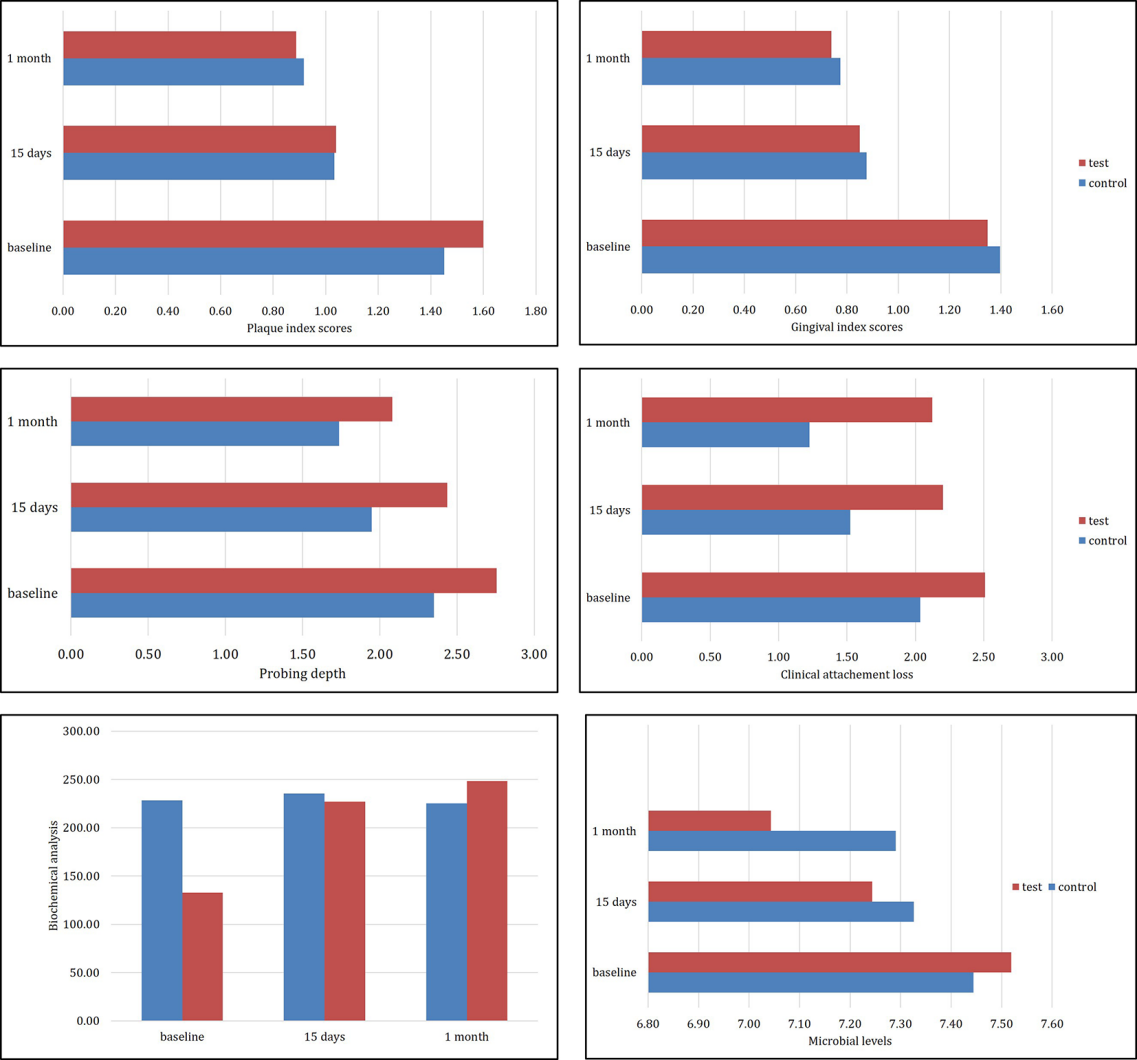
Changes in gingival index, plaque index, bleeding on probing, pocket depth and clinical attachment, microbiologic colony-forming units, and antioxidant levels of protein thiol variables at each recall visit.

The study results showed that the mean age of study participants in the test group was 35.42 ± 11.79 years and in the controls was 32.12 ± 12.85. The gender wise distribution in the test group was Males: 16; Female: 14 and Control group was Males: 13; Females: 14 ( Table 2). The comparison between goji berry mouth group and chlorhexidine group for of all the study variables was found to have no significant differences in the mean values for Pi (P = 0.470), Gi (P = 0.239), BOP (P = 0.450), PPD (P = 0.216), CAL (P = 0.220), and Microbial level (P = 0.251) ( Table 3). The intra-group comparison using the ‘repeated measures of ANOVA with Greenhouse Geisser correction’ followed by a ‘post-hoc analysis with Bonferroni adjustment’. This showed that the mean PPD, Pi, and Gi, was changed from baseline to one month in both the control and test groups. The CAL was reduced significantly only in the chlorhexidine group compared to the goji berry group ( Table 3, Table 4 and Figure 3). A significant difference was noted in the antioxidant levels (protein thiol) in saliva in the goji berry group at the end of one month. No change in the salivary antioxidant level was noted in the chlorhexidine group. No significant differences were reported in the log-transformed microbial CFU counts in both groups at any given time. In the case of the test group, the mean Pi reduced from 1.6 ± 0.38 at baseline and was 0.89 ± 0.17 at one month. However, in the case of the goji berry mouthwash, the mean PPD was reduced during the 15-day follow-up (1.73 ± 0.45) compared to baseline (2.76 ± 1.06). The intergroup comparison at one-month follow-up (15 days and one month) after adjusting the respective baseline scores was done using ANCOVA. The control group had a significant reduction in CAL (P = 0.001) as compared to the test group. There were no differences seen in the mean values for Pi (P = 0.791), Gi (P = 0.594), PPD (P = 0.134), protein thiol levels (P = 0.211), and microbial levels (P = 0.188) between the two groups ( Table 4). No harms were reported by any patient. One patient reported bitter taste of the mouthwash and discontinued the mouthwash.

**Table 2.** Demographic data of the groups.

Groups	Goji berry group (case group)	Chlorhexidine group (Control group)	p-value
**Age (in Years)**	35.42 ± 11.79	32.12 ± 12.85	0.29 ^a^
	**Male-Female**	**Male-Female**	
**Gender**	16-14	13-14	0.11 ^b^

Based on: One-way ANOVA/Chi Square test.

**Table 3.** Inter-group and intra-group comparison of baseline, 15 days, and 1-month follow-up scores.

	Group	Baseline	15 days	1 month	P-value ¥		
		Mean ± SD	Mean ± SD	Mean ± SD	Baseline vs 15 days	Baseline vs 1 month	15 days vs 1 month
**Plaque index**	**Control**	1.45 ± 0.42	1.03 ± 0.50	0.92 ± 0.43	0.104	**0.027**	0.334
	**Test**	1.6 ± 0.38	1.04 ± 0.36	0.89 ± 0.17	**0.000**	**0.000**	0.076
	**P-value** **#**	0.470					
**Gingival index**	**Control**	1.4 ± 0.64	0.87 ± 0.47	0.81 ± 0.43	**0.001**	**0.036**	0.214
	**Test**	1.35 ± 0.38	0.85 ± 0.31	0.75 ± 0.24	**0.000**	**0.000**	0.092
	**P-value** **#**	0.239					
**Percentage of sites with bleeding on probing**	**Control**	80.7 ± 0.42	42.7 ± 0.49	10.7 ± 0.42	**0.001**	**0.034**	0.210
	**Test**	84.8± 0.32	40.8± 0.29	09.0± 0.32	**0.000**	**0.000**	0.090
	**P-value** **#**	0.450					
**Probing pocket depth**	**Control**	2.35 ± 0.56	1.62 ± 0.56	1.78 ± 0.35	**0.004**	**0.006**	0.186
	**Test**	2.76 ± 1.06	1.73 ± 0.45	2.15 ± 0.68	**0.002**	**0.035**	0.327
	**P-value** **#**	0.216					
**Clinical attachment level**	**Control**	2.03 ±0.90	1.52 ± 0.65	1.33 ± 0.43	**0.020**	**0.005**	0.174
	**Test**	2.62 ± 1.16	2.29 ± 0.99	2.02 ± 0.61	**0.103**	0.248	0.260
	**P-value** **#**	0.220					
**Biochemical analysis**	**Control**	233.06 ± 144.49	235.48 ± 103.39	225.06 ± 72.11	0.874	0.899	0.760
	**Test**	132.68 ± 56.17	227.05 ± 72.21	248.30 ± 68.31	**0.000**	**0.000**	0.272
	**P-value** **#**	**0.021**					
**Microbial level**	**Control**	7.44 ± 0.43	7.35 ± 0.584	7.33 ± 0.567	0.183	0.130	0.756
	**Test**	7.57 ± 0.77	7.19 ± 0.74	7.04 ± 0.85	0.222	0.564	0.264
	**P-value** **#**	0.251					

Test: L. barbarum mouthwash; Control: chlorhexidine gluconate mouthwash.

N for Case group = 15; Control group = 14. Values in bold indicate significant difference.

^#^ P-value for inter-group comparisons (independent sample *t-test*);

^¥^ P-value for intra-group comparisons (Repeated measures ANOVA).

**Table 4.** Inter-group comparisons at 1-month follow-up after adjusting for baseline values.

Outcomes	Adjusted baseline	I month		P-value
		Control Mean ± SE	Test Mean ± SE	
**Plaque index**	1.61	0.92±.092	0.88±0.09	0.791
**Gingival index**	1.46	0.82±0.100	0.75±0.10	0.594
**Percentage of sites with BOP**	78.4	12.9 ±0.56	13.1 ±0.42	0.494
**Probing pocket depth**	2.06	1.77±0.254	2.15±0.26	0.134
**Clinical attachment level**	2.33	1.33±1.22	2.02±0.11	**0.001**
**Biochemical analysis**	182.77	225.06 ±16.83	248.30 ±16.83	0.211
**Microbial level**	7.45	7.33 ±0.17	7.05 ±0.19	0.188

Values in bold indicate significant difference.

## Discussion

The present study is the first clinical trial aimed to evaluate the role of goji berry in treating any dental diseases. The study evaluated the effectiveness of goji berry mouthwash compared to chlorhexidine for managing chronic periodontitis in both males and female patients. Based on the results, it was noted that goji berry mouthwash can control the gingival inflammation, BOP, and plaque formation at 15 days following SRP with statistically significant difference compared to chlorhexidine group. A significant in the Gi, Pi, BOP, PPD was noted in participants using goji berry mouthwash. To our knowledge, this is first clinical trial comparing the potential of *L. barbarum* and chlorhexidine (gold standard antimicrobial agent) as an adjunct to SRP for the management of chronic periodontitis. The most significant outcome was the statistically significant increase in the protein thiol levels in saliva in the goji berry group alone, suggesting the advantage of using the goji berry mouthwash over chlorhexidine *.* This could be attributed to powerful antioxidants in goji berry mouthwash compared to chlorhexidine.

Goji berry is considered a ‘superfood’ with good antimicrobial, anti-inflammatory, and antioxidant properties. The constituents of *L. barbarum* have been tried for various inflammatory and infectious disease, however its role in treating periodontal inflammation has not been explored. The use of *L. barbarum* could improve the local antioxidant levels and this in turn helps in controlling the clinical symptoms of inflammation in chronic periodontitis. The study is crucial, especially with the emergence of resistance to chlorhexidine molecules among oral bacteria and numerous side effects of using chlorhexidine for long time. ^51^ Recent systematic reviews have shown that there are limited benefits of using chlorhexidine gluconate in patients with chronic periodontitis owing to the development of resistance among oral bacteria to chlorhexidine molecule and cytotoxicity to soft tissues by chlorhexidine. ^52^ Hence the use of plant-based products with antioxidant and anti-inflammatory properties is advocated to control the local oxidative stress in the gingival tissues and preclude the harmful effects of chlorhexidine. ^53^

Goji berry has numerous antioxidants, which could be utilized to combat the proinflammatory mediators and oxidative stress in the periodontal tissues. ^54^^–^^56^ Some of the important antioxidants in *L. barbarum* are the *Lycium barbarum* polysaccharides (LBPs), caffeic acid, coumaric acid, scopoletin, linoleic acid, zeaxanthin dipalmitate, kaempferol, caffeoylquinic and coumaric acid. ^55^^–^^57^ Studies have also identified different types of flavonoids such as catechin, epicatechin, and quercetin in goji berries. Flavonoids have a good antimicrobial effect on the various periodontal pathogen and this could justify the reduction in the PI and control the gingival inflammation in participants using goji berry mouthwash. The alcoholic group (-OH) in the goji berry is also another antimicrobial agent that can control the microbial growth in oral biofilm. ^58^ Goji berry also contains monoterpenes (like sabinene, phellandrene, γ-terpinene), phenolic acid (chlorogenic acid), citric acid, tartaric acid, oxalic acid, malic acid, and vitamin C. These compounds have shown superior anti-inflammatory and antibacterial properties. ^59^ LBP in goji berry is also a potent inhibitor of proinflammatory cytokines. It can inhibit matrix metalloproteinase (MMP-1), Tumor necrosis factor, Interleukin-2, and PGE2. This can helps to control connective tissue destruction, which is a crucial aspect in the progression of periodontal disease. Goji berries can help to improve fibroblast healing and increase the production of Type I collagen. ^41^^,^^60^ This can be attributed to the ability to reduce gingival inflammation and release of various antioxidants. Chlorogenic acid in the goji berry can even inhibit nitric oxide and inducible nitric oxide synthase which are some of the vital pathways activated during periodontal inflammation. ^61^^–^^63^ At the molecular level, the polysaccharides in the goji berries (LBP) influence the ‘antigen-presenting cells’ such as dendritic cell. This improves the adaptive immune response at the site of inflammation and control tissue destruction. ^61^ Direct effect of chlorogenic acid, yet another important constituent in goji berry, on the oxidative stress-induced secretion of proinflammatory cytokines and mRNA expression has also been proven. ^62^^,^^63^

Based on our results and exitsing evidence, it can be concluded that goji berry can be an effective adjunct to oral prophylaxis compared to chlorhexidine in patients with chronic periodontitis. ^64^^,^^65^ Although our study has proven that goji berry mouthwash is an effective adjunct to SRP for managing periodontitis, future studies should compare the efficacy of goji berry with other herbal or plant-based adjuncts with long term follow up. One should also note that the study tested the role of goji berry for only a short recall time, as prolonged exposure to chlorhexidine mouthwash is associated with many side effects. Future studies can explore the effect of goji berry on longer recall visits. Future studies can also evaluate the effect of *L. barbarum* on specific periodontal pathogens such as red complex bacteria or *P. gingivalis* or specific biomarkers for periodontitis.

## Conclusion

*L. barbarum* is a promising ‘superfood’ with many potential health benefits. It has antioxidant and antimicrobial properties that can be utilized for developing novel formulations for treating patients with chronic periodontitis. *L. barbarum* mouthwash effectively reduced plaque scores, gingival inflammation, and bleeding on probing. Goji berry can be tried instead of chlorhexidine gluconate for managing periodontal disease.

### Ethical statement

The study was conducted after receiving ethical approval from Kasturba Medical College and Kasturba Hospital Ethic committee with IEC no: IEC no 117/2019. The trial has been registered at the ‘Clinical trial registry (CTRI/2019/05/019042)’.

## Data Availability

Figshare. Data on study titled: Lycium barbarum (Goji berry) mouthwash is a viable alternative to 0.2% chlorhexidine gluconate for managing chronic periodontitis: a randomized clinical trial. DOI: https://doi.org/10.6084/m9.figshare.21834939.v1. ^65^ This project contains the following data:
- CONSORT checklist - Study protocol Figshare. Goji berry mouthwash as alternative to chlorhexidine for managing chronic periodontitis. DOI: https://doi.org/10.6084/m9.figshare.21780164.v1. ^64^ This project contains the following data:
- Study data Data are available under the terms of the Creative Commons Attribution 4.0 International license (CC-BY 4.0). The datasets related to our study is also available with the corresponding author and can be shared on reasonable request via email to aditi.chopra@manipal.edu.
