# *Efficacy of Lycium barbarum* (Goji berry) mouthwash for managing periodontitis: a randomized clinical trial

**DOI:** 10.12688/f1000research.129891.4

**Published:** 2024-11-26

**Authors:** Amee Sanghavi, Laasya Shettigar, Aditi Chopra, Ashmeet Shah, Richard Lobo, Padmaja A Shenoy, ShivaPrasada Gadag, Usha Y Nayak, Mangalore Shravya S, Shobha Ullas Kamath, Prajna P Nayak

**Affiliations:** 1 Manipal College of Dental Sciences, Manipal Academy of Higher Education, Manipal, Karnataka, 576104, India; 2 Periodontology, Manipal College of Dental Sciences, Manipal Academy of Higher Education, Manipal, Karnataka, 576104, India; 3 Department of Pharmacognosy, Manipal College of Pharamcuetical Sciences, Manipal Academy of Higher Education, Manipal, Karnataka, 576104, India; 4 Microbiology, Kasturba Medical College and Hospital, Manipal Academy of Higher Education, Manipal, Karnataka, 576104, India; 5 Pharmacuetics, Manipal College of Pharmacuetical Sciences, Manipal Academy of Higher Education, Manipal, Karnataka, 576104, India; 6 Biochemistry, Kasturba Medical College and Hospital, Manipal Academy of Higher Education, Manipal, Karnataka, 576104, India; 7 Public Health Dentistry, Manipal College of Dental Sciences, Manipal Academy of Higher Education, Manipal, Karnataka, 576104, India

**Keywords:** Periodontitis, Periodontal disease, Oral health, Dental Hygiene, Lycium Barbarum, Goji berry, Mouthwash, Chlorhexidine, Herbal, Antioxidants

## Abstract

**Background:**

Removal of the microbial deposits (plaque and calculus) by performing effective scaling and root planing (SRP) is the fundamental step for managing periodontal disease (gingivitis and periodontitis). Various adjuncts in the form of mouthwash, gels, and toothpaste are also being used as adjuncts to SRP for managing periodontitis. Recently, *Lycium barbarum* ( *L. barbarum*), commonly known as goji berry or wolfberry, has gained popularity for managing chronic inflammatory and infectious diseases. However, its efficacy in managing periodontitis has never been explored. Hence the present study aims to evaluate the efficacy of goji berry mouthwash compared to chlorhexidine mouthwash for managing periodontitis.

**Methods:**

60 adult participants were divided randomly using computer-generated random sequences into two groups (case group: *L. barbarum* mouthwash (Males: 16; Female: 14); control group: 0.2% chlorhexidine gluconate mouthwash (Males: 14; Females: 16)). The changes in the gingival index (Gi), plaque index (Pi), bleeding on probing (BOP), clinical attachment loss (CAL), probing pocket depth (PPD), microbial load, and antioxidant levels (protein thiol) in saliva were noted at the baseline, at 15 days and one month.

**Results:**

A significant reduction in the mean PPD, Pi, BOP, and Gi was seen from baseline to one month in both the control (P-value=0.006, 0.027, 0.000, and 0.036 respectively) and test groups (P-value=0.035, 0.000, 0.034, and 0.000 respectively). However, the antioxidant levels (protein thiol) in saliva were significantly increased only in the test group.

**Conclusion:**

Goji berry mouthwash along with SRP reduced the Gi, Pi, BOP, and PPD in patients with periodontitis. However, no statistically significant difference was noted between the use of goji berry and chlorhexidine mouthwash. Goji berry mouthwash was more effective than chlorhexidine in increasing the antioxidant levels in saliva.

## Introduction

Periodontitis is defined as a chronic immuno-inflammatory multifactorial disease that affects the soft tissue around the teeth. ^1^ Severe periodontitis and edentulism represent a significant public health challenge, with the number of affected individuals projected to increase considerably in the coming decades. In 2021, over one billion people were affected by severe periodontitis, with a global age-standardised prevalence of 12.50%. South Asia had by far the highest prevalence rate, at 17.57%. It may be reasonably extrapolated that severe periodontitis will be among the most impactful level 4 diseases/conditions affecting years lived with disability worldwide by 2050. ^2^ It is more prevalent in males compared to females. It is primarily caused by the interaction of the oral microorganisms with the host (soft tissues surrounding the teeth. This host-microbial interaction triggers a massive influx of various pro-inflammatory mediators, microbial by-products, proteolytic enzymes, and free reactive oxygen species (ROS) resulting in increased periodontal hard and soft tissue destruction and inflammation. ^3^^–^^6^ Due to increased ROS during periodontal disease, it is also referred to as free radical-mediated tissue injury. ^7^ Some of the common putative periodontal microbes associated with periodontitis are *Porphyromonas gingivalis, Tanerella forsythia, Treponema denticola, Campylobacter rectus,* and *Fusobacterium nucleatum.* ^7^ Apart from the microbial etiology, other risk factors such as smoking, diabetes mellitus, HIV, nutritional deficiency, medications, poor oral hygiene, and genetics influence the severity of periodontal inflammation. ^8^

To control periodontal inflammation the primary and most vital step is the removal of oral biofilm formed around the teeth by performing effective mechanical debridement with either hand or machine-driven instruments. ^9^^,^^10^ Effective scaling and root planing (SRP) can reduce gingival inflammation, thereby preventing disease progression and restoring gingival health. ^11^ However, studies have shown that complete debridement of calculus is technically demanding, as many times removal of hard and soft tissue deposits from deep pockets (>5 mm) and interdental regions becomes challenging. ^11^^,^^12^ Additionally, it is difficult to completely remove the smooth or burnished calculus from deep and circuitous periodontal pockets, furcation areas, root concavities, and irregular roots owing to a lack of good visibility and accessibility to such areas. ^12^^,^^13^ Hence for the management of biofilm from deep and tortuous pockets additional periodontal therapy and use of adjuncts are needed. ^12^^–^^15^

It is also noted that the efficacy of SRP is dependent on the patient’s compliance and motivation to maintain a meticulous oral care regime and effective plaque control at home. ^16^ SRP alone may not be sufficient to maintain the required plaque control if patient compliance is poor and the patient does not effectively follow oral hygiene instructions. Additionally, studies have shown that even after a good plaque control regime; posterior, palatal, and lingual surfaces of the teeth retain some amount of plaque. Thus, for many patients, adjuncts such as mouthwash and gels along with regular toothbrushing are indicated. ^11^^,^^17^^–^^24^ Various chemical plaque control agents with antimicrobial and anti-inflammatory are being used in the form of mouthwash, gels, gum paints, fibers, varnishes, microspheres, chips, tablets, powder, and capsules for managing gingivitis and periodontitis. Among these agents, chlorhexidine gluconate is the most popular and routinely used agent for managing periodontitis. Additionally, recent reports have shown no additional benefits of using chlorhexidine and the development of antimicrobial resistance among many oral bacteria to chlorhexidine molecules. ^25^^–^^29^ Prolonged use of chlorhexidine is contraindicated owing to various side effects like altered taste sensation, staining of the teeth and soft tissues such as tongue and mucosa, increased calculus formation, and parotid gland swellings. ^25^ Chlorhexidine is also known to have cytotoxic effects on the gingival fibroblasts, ^26^ periodontal ligament, ^27^ and osteoblastic cells. ^28^

Thus, there is an emerging trend to use natural and herbal extracts with antioxidant and antimicrobial properties to treat periodontal diseases. Herbal extracts from neem, Tulsi, guava, green tea, turmeric, curcumin, pomegranate, and many more plants have been tried to effectively treat gingivitis and periodontitis. ^30^^–^^34^ Since periodontal disease causes a massive release of ROS and increases oxidative stress, adjuncts with antioxidant potential are often used with SRP to control the oxidative stress present locally in the gingival and periodontal tissues. Recently, goji berry, commonly known as Wolfberry, Himalayan goji, or Tibetan goji, has gained a lot of popularity due to its strong antioxidant and anti-inflammatory properties. ^35^^–^^46^

Goji berry, scientifically known as *Lycium barbarum (L. barbarum)*, is a fruit native to southeast Europe, China, and Asia. ^35^ The fruit belongs to the family of Solanaceae and is consumed in both fresh and dried forms. It has powerful antioxidant, antimicrobial, immuno-modulating, and anticancer properties. ^36^^–^^46^ It is used for the treatment of many inflammatory and infectious diseases, including oral diseases. ^36^^–^^46^ Soesanto et al. (2021) showed that ethanolic extract of *L. barbarum* is effective against oral bacteria ( *Streptococcus mutans* and *P. gingivalis*) at 100 μg/mL. ^47^ Previous *in-vitro* studies have also reported that the minimal inhibitory concentration (MIC) of *L. barbarum* was comparable to chlorhexidine for inhibiting oral bacteria, however, its efficacy was less as compared to the antibiotic doxycycline. At 50 μg/mL, ethanolic extract of goji berry could inhibit most of the periodontal pathogens. ^48^^,^^49^ However, no clinical study has yet assessed the effectiveness of goji berry mouthwash as an adjunct to SRP for the management of periodontitis. Therefore, this clinical study aims to evaluate the efficacy of *L. barbarum* mouthwash along with SRP for patients with periodontitis compared to chlorhexidine for the first time.

### The objectives of the study include

1. To evaluate the effect of *L. barbarum* (goji berry) mouthwash on the Plaque index (Pi), Gingival index (Gi), Bleeding on probing (BOP), probing pocket depth (PPD), and clinical attachment loss (CAL) at baseline, 15 days and one month compared to 0.2% chlorhexidine gluconate mouthwash.
2. To evaluate and compare the change in the protein thiol levels in saliva at 15 days, and one month compared to baseline in participants using goji berry and chlorhexidine mouthwash.
3. To evaluate and compare the reduction in the microbial count at the end of one month compared to baseline in participants using *L. barbarum* mouthwash compared to chlorhexidine mouthwash.

## Methods

### Trial design

**Figure 1.**
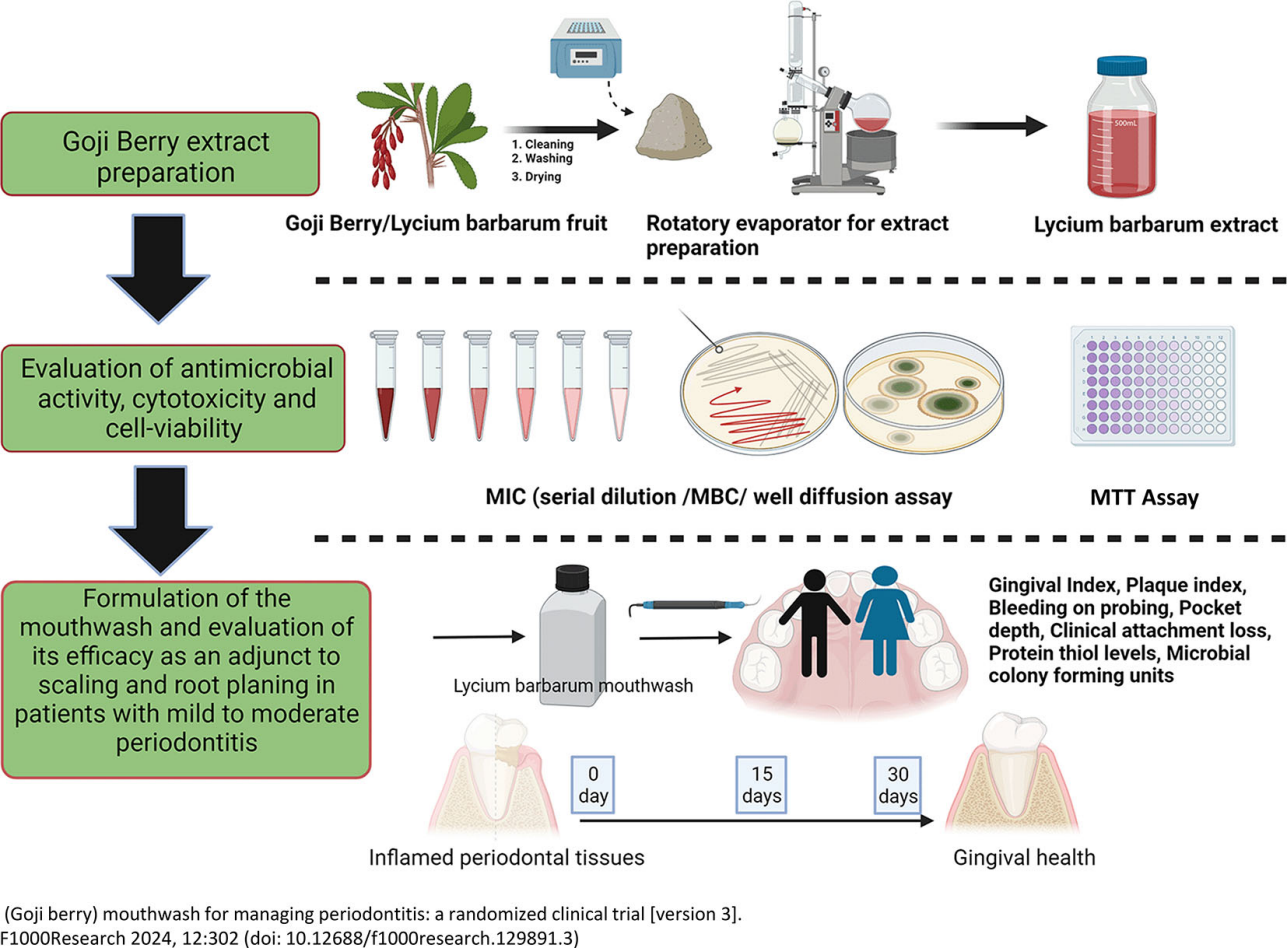
Schematic representation of the study design (Created in Biorender.com).

**Figure 2.**
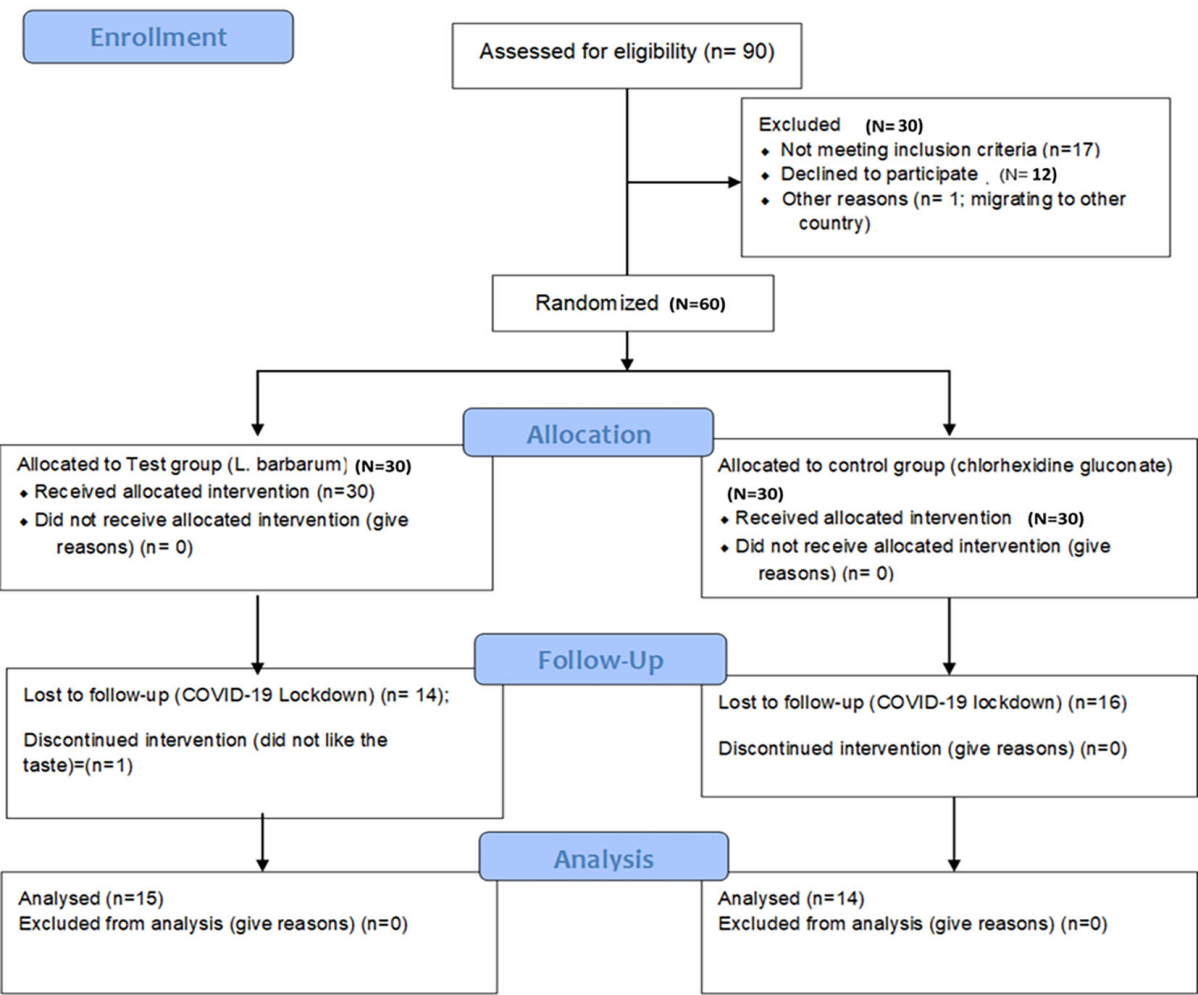
Participant flow diagram.

The study was designed as a randomized, double-blind single-centered parallel arm clinical trial with an allocation ratio of 1:1. The study was conducted at Manipal’ from 2019 to 2020 following the “Helsinki Declaration of 1975 (as revised in 2000)”. The trial was initiated after receiving ethical clearance from the Kasturba Medical Hospital Institutional Ethics Committee with IEC no 117/2019. The trial was registered at the ‘Clinical Trial Registry (CTRI/2019/05/019042)’ and followed the CONSORT and SAGER guidelines ( Figures 1 and 2). ^48^ The steps in the clinical trial are explained as follows:

#### Formulation of mouthwash

The mouthwash was prepared at the “Manipal College of Pharmaceutical Sciences, Manipal”. *L. barbarum* mouthwash was prepared by preparing an ethanolic extract of dried goji berry using Soxhlet’s method and dissolving the 50 ug/ml of weighed goji berry extract for formulating the mouthwash as described previously. ^49^ A concentration of 50 ug/ml was mixed in 1.15 of 100% alcohol (ethanol) and then homogenized using 15% glycerol. To this around 15% propylene glycol and 1% Tween solution was added to formulate the mouthwash. 0.1% menthol was added to adjust the taste of the mouthwash. The volume was adjusted to 100% by using distilled water. 100% alcohol was added to serve both as a preservative and a dissolvent.

#### Assessment of stability of mouthwash

The shelf life of the mouthwash was measured as per the ICH guidelines and the samples were tested for three months under three different conditions: “35°±2% C with a relative humidity of 60% ± 5%; 25°C± 2°C and relative humidity: 60% ± 5%; and 40°C ± 2°C with a relative humidity of 75% ± 5%”. The mouthwash was kept in opaque plastic bottles and one of the bottles was kept in the stability compartment (Thermo lab, India). The samples were analyzed at baseline, one, two, and three months by visual observation and UV spectrum analysis (Shimadzu UV-1601PC, Japan) and analyzed for their physical parameters and stability.

#### Clinical study design

Ninety subjects aged 20 to 50 years (both male/females) visiting the outpatient department were screened for the presence of localized/generalized periodontitis according to the 2017 classification of periodontal disease and selected based on the following exclusion and inclusion criteria ^50^:

***Inclusion criteria:***

1. Participants in the age group of 20-50 years diagnosed with stage I to stage II localized/generalized periodontitis (Grades A to B).
2. Participants with a minimum of 24 functional teeth.

***Exclusion criteria:***

1. Participants with any allergic reactions to chlorhexidine.
2. Participants with any systemic diseases such as hypertension, diabetes mellitus, cardiovascular, renal, and neurological diseases.
3. Participants who were on any anti-inflammatory, antibiotics, or analgesics in the last six months.
4. Pregnant or lactating mothers.
5. Participants with any oral abusive habits such as smoking, alcohol, betel nut chewing, gutka, paan, supari, and areca nut.
6. Participants who had undergone any periodontal treatment/surgery in the last six months.
7. Participants undergoing orthodontic treatment.
8. Participants using any other oral hygiene agents (mouthwash or gels) were excluded to remove confounding bias arising due to the difference in plaque control measures.

All participants who satisfied the above-mentioned criteria were recruited after obtaining both oral and written informed consent.

### Randomization, allocation concealment, and blinding

After screening the participants, 60 participants were recruited. The participants were randomly allocated to the test and control group using the computer-generated random sequence as follows: the test group ( *L. barbarum*, n = 30) or the control group (0.2% chlorhexidine gluconate mouthwash (Hexidinie, ICPA Health Products, India) n = 30). This sample size was based on p-value <0.05, alpha value = 0.05 two-tailed, power = 0.8, and the effect size = 0.7 for CAL. The allocation of participants into two groups was done by an investigator who was not part of either biological sample collection, analysis, periodontal therapy (SRP), or clinical or periodontal examination. The allocation concealment was done by dispensing both mouthwash in similar-sized opaque amber-colored bottles to all participants. The investigators giving the bottles to the patients did not know the content and nature of the mouthwash. The participants were also blinded about their group about the content of the mouthwash, although the taste of the mouthwash could not be matched. The investigator and statistician analyzing the clinical data were not aware of the grouping. Following grouping, the following biological samples were collected at baseline:

***a. Microbiology plaque sample collection***

“The plaque from the subgingival region was collected from the pocket with the greatest probing depth in each quadrant using a sterile, site-specific Gracey curette. Following collection, the curette was promptly immersed in a reduced transport medium (Thioglycolate both supplemented with hemin and vitamin K) for microbiological analysis. Each sample underwent vortexing, and quantitative cultures were performed across various dilutions (1:10, 1:100, 1:1000, and 1:10,000). A volume of 10 μl from each dilution was plated onto pre-reduced 5% sheep blood agar (BD, Becton Dickinson, Heidelberg, Germany), with plates incubated at 37°C for 48 hours in an anaerobic chamber (Whitley A35 Anaerobic workstation, Don Whitley Scientific, Shipley, UK). The colony-forming units were analyzed for each sample using the following formula: CFU/mL = (number of colonies × dilution factor)/volume of the culture plate.”

***b. Saliva Collection***

Following plaque collection, a stringent method for saliva collection was followed. All participants were requested to sit comfortably in an upright position. Following this, around 2ml of saliva was collected by the ‘spitting method’ and without any stimulation. ^51^ All participants were requested to spit saliva into an Eppendorf vial. The collected saliva was then stored immediately in a refrigerator at -80 degrees Celsius. The Eppendorf tubes used to store saliva samples were numerically marked according to the participant number. The saliva collected was sent for biochemical analysis for evaluation of the protein thiol levels using Ellman’s Reagent. The absorbance of the agent was measured after incubation at room temperature at 412 nm for around five minutes and the concentration of protein thiol was determined with the standard curve of glutathione.

***c. Examination of the clinical periodontal parameters***

Following saliva sample collection, a periodontal examination was done for all the sextants for all participants. The following periodontal parameters were recorded: Gi by Loe & Silness, 1963; percentage of sites with BOP; Pi by Silness & Loe 1964; PD and CAL. All the clinical evaluations were done by an investigator who was blinded about the patient’s grouping. The BOP, PPD, and CAL were recorded by the Williams periodontal probe (Hu-Friedy, USA). The sites with BOP were checked by noting the presence and absence of bleeding on all four surfaces (buccal, lingual, mesial, and distal) for all the teeth. The percentage of sites with BOP was considered by the percentage of the teeth with BOP to the total teeth present. The PPD and CAL were calculated at the buccal, mesial, distal, and lingual sides. At the interdental region (mesial and distal), probing was done from both the buccal and lingual sides. The deepest pocket depth at each surface was recorded. The average or mean of the interdental pocket on each side along with buccal and lingual probing depth was considered as the final PD for that tooth. The mean PPD and CAL were assessed by adding the reading from each tooth and dividing it by the total number of teeth.

A thorough SRP was initiated for all the participants after periodontal examination and sample collection. A single trained investigator examined the baseline and follow-up visits for all the patients. The supervisor assessed the scaling to ensure the complete removal of plaque and calculus was done. All patients were educated to brush their/teeth in modified bass technique for two minutes twice daily, nullifying any confounding effects arising from differences in the oral hygiene measures. All participants were given the opaque amber color bottles which were coded (AX or BX). This was done to blind the patients and investigators regarding the type of mouthwash given to the participants. All patients were instructed to use 10 ml mouthwash (goji berry mouthwash) diluted with 10 ml of water for 30-45 seconds twice a day for a month. For chlorhexidine (Hexidine mouthwash, India), participants were requested to use the undiluted mouthwash. Patients were recalled after 15 days and one month for revaluation. At each recall visit Pi, Gi, percentage of sites with BOP, CAL, and PPD were noted clinically. The plaque from the same site and saliva samples were also collected at each recall visit. The investigation for each participant, at each recall visit, was done by the same investigator.

### Statistical analysis

Data obtained was analyzed by the ‘SPSS version 26.0, IBM’. For descriptive data frequency and categorical data mean and standard deviation for all the numerical data were analysed using the ‘Kolmogorov–Smirnov test’. The normality of the distribution was checked for all variables. The inter-group comparisons of all the assessed outcomes were done using an ‘independent sample t-test’. The comparisons between the goji berry mouthwash and chlorhexidine mouthwash were done to measure any significant increase/reduction from baseline to 15 days and one month using Repeated measures ANOVA with Geisser correction’ followed by a ‘post-hoc analysis with Bonferroni adjustment’. Inter-group comparison of reduction of all the variables at follow-up was done using ANCOVA after adjusting the respective baseline scores. The p-value of less than 0.05 was considered to be significant.

## Results

The results of the stability of the mouthwash showed no change in color, odor, consistency, or phase separation at three months. The sample showed no change when observed under the ultraviolet analysis at 274 nm. This indicated that the mouthwash was stable till the end of three months ( Table 1).

**Table 1.** Physical properties and evaluation of the stability of the mouthwash.

Temperature	Evaluation parameters	Observation (in months)			
		Baseline	1	2	3
** Room temperature (3 – 5 ± 2%°C)**	**Visual appearance**	Slight yellowish-brown	Slight yellowish-brown	Slight yellowish-brown	Slight yellowish-brown
	**Precipitation/Phase separation**	Nil	Nil	Nil	Nil
	**Homogeneity**	Good	Good	Good	Good
**Room temperature (25°C ± 2°C) Relative humidity (60% ± 5%)**	**Visual appearance**	Slight yellowish-brown	Slight yellowish-brown	Slight yellowish-brown	Slight yellowish-brown
	**Precipitation/Phase separation**	Nil	Nil	Nil	Nil
	**Homogeneity**	Good	Good	Good	Good
**Room temperature 40°C ± 2°C Relative humidity (75% ± 5%)**	**Visual appearance**	Slight yellowish-brown	Slight yellowish-brown	Slight yellowish-brown	Slight yellowish-brown
	**Precipitation/Phase separation**	Nil	Nil	Nil	Nil
	**Homogeneity**	Good	Good	Good	Good

Results from the clinical trial: Of the 90 participants assessed for eligibility, 30 participants were excluded as they did not meet the eligibility criteria. The rest 60 participants were allocated into test (n=30) and control (n=30) groups. The mean age of participants in the test group was 35.42 ± 11.79 years and in the controls was 32.12 ± 12.85. The gender-wise distribution in the test group was males: 16; females: 14 and the control group was males: 14; females: 16 ( Table 2). Out of the 30 patients in each group, 14 patients in the test group and 16 patients in the control did not come for the follow-up visit due to the sudden lockdown imposed by COVID-19. Additionally, one patient in the test group reported the bitter taste of the goji berry mouthwash and discontinued the mouthwash. Thus, for analysis 15 patients in the test and 14 patients in the control were included in the analysis. The comparison between goji berry mouthwash and chlorhexidine mouthwash showed no significant differences in the mean values for Pi (p = 0.470), Gi (p = 0.239), BOP (p = 0.450), PPD (p = 0.216), CAL (p = 0.220), and Microbial level (p = 0.251) ( Table 3).

**Table 2.** Demographic data of the groups.

Groups	Goji berry group (case group)	Chlorhexidine group (Control group)	p-value
**Age (in Years)**	35.42 ± 11.79	32.12 ± 12.85	0.29 ^a^
	**Male-Female **	**Male-Female **	
**Gender**	16-14	13-14	0.11 ^b^

Based on: One-way ANOVA/Chi Square test.

**Table 3.** Inter-group and intra-group comparison of baseline, 15 days, and 1-month follow-up scores.

	Group	Baseline	15 days	1 month	P-value ¥		
		Mean ± SD	Mean ± SD	Mean ± SD	Baseline vs 15 days	Baseline vs 1 month	15 days vs 1 month
** Plaque index**	** Control**	1.45 ± 0.42	1.03 ± 0.50	0.92 ± 0.43	0.104	** 0.027**	0.334
	**Test**	1.6 ± 0.38	1.04 ± 0.36	0.89 ± 0.17	**0.000**	**0.000**	0.076
	**P-value ** **#**	0.470					
**Gingival index**	**Control**	1.4 ± 0.64	0.87 ± 0.47	0.81 ± 0.43	**0.001**	**0.036**	0.214
	**Test**	1.35 ± 0.38	0.85 ± 0.31	0.75 ± 0.24	**0.000**	**0.000**	0.092
	**P-value ** **#**	0.239					
**Percentage of sites with bleeding on probing**	**Control**	80.7 ± 0.42	42.7 ± 0.49	10.7 ± 0.42	**0.001**	**0.034**	0.210
	**Test**	84.8± 0.32	40.8± 0.29	09.0± 0.32	**0.000**	**0.000**	0.090
	**P-value ** **#**	0.450					
**Probing pocket depth**	**Control**	2.35 ± 0.56	1.62 ± 0.56	1.78 ± 0.35	**0.004**	**0.006**	0.186
	**Test**	2.76 ± 1.06	1.73 ± 0.45	2.15 ± 0.68	**0.002**	**0.035**	0.327
	**P-value ** **#**	0.216					
**Clinical attachment level**	**Control**	2.03 ±0.90	1.52 ± 0.65	1.33 ± 0.43	**0.020**	**0.005**	0.174
	**Test**	2.62 ± 1.16	2.29 ± 0.99	2.02 ± 0.61	**0.103**	0.248	0.260
	**P-value ** **#**	0.220					
**Biochemical analysis**	**Control**	233.06 ± 144.49	235.48 ± 103.39	225.06 ± 72.11	0.874	0.899	0.760
	**Test**	132.68 ± 56.17	227.05 ± 72.21	248.30 ± 68.31	**0.000**	**0.000**	0.272
	**P-value ** **#**	**0.021**					
**Microbial level**	**Control**	7.44 ± 0.43	7.35 ± 0.584	7.33 ± 0.567	0.183	0.130	0.756
	**Test**	7.57 ± 0.77	7.19 ± 0.74	7.04 ± 0.85	0.222	0.564	0.264
	**P-value ** **#**	0.251					

Test: L. barbarum mouthwash; Control: chlorhexidine gluconate mouthwash.

N for Case group = 15; Control group = 14. Values in bold indicate significant difference.

^#^ P-value for inter-group comparisons (independent sample *t-test *);

^¥^ P-value for intra-group comparisons (Repeated measures ANOVA).

The intra-group comparison showed a significant reduction in the mean PPD, Pi, BOP, and Gi, and antioxidant capacity from baseline to one month in both the control and test groups. The CAL reduced significantly only in the chlorhexidine group compared to the goji berry group ( Table 3, Table 4). The percentage of sites with BOP reduced from 84.8 ± 0.32 at baseline in the test group to 09.0 ± 0.32 at the end of six months. These results were comparable to the control group with no statistically significant difference between the test and control. A significant difference was noted in the antioxidant levels (protein thiol) in saliva in the goji berry group alone at the end of one month. No change in the salivary antioxidant level was noted in the chlorhexidine group. No significant differences were reported in the log-transformed microbial CFU counts in both groups at any given time. In the case of the test group, the mean Pi reduced from 1.6 ± 0.38 at baseline and was 0.89 ± 0.17 at one month. However, in the case of the goji berry mouthwash, the mean PPD was reduced during the 15-day follow-up (1.73 ± 0.45) compared to the baseline (2.76 ± 1.06).

**Table 4.** Inter-group comparisons at one month follow-up after adjusting for baseline values.

Outcomes	Adjusted baseline	One-month		p-value
		Control Mean ± SE	Test Mean ± SE	
**Plaque index**	1.61	0.92±.092	0.88±0.09	0.791
**Gingival index**	1.46	0.82±0.100	0.75±0.10	0.594
**Percentage of sites with BOP**	78.4	12.9 ±0.56	13.1 ±0.42	0.494
**Probing pocket depth**	2.06	1.77±0.254	2.15±0.26	0.134
**Clinical attachment level**	2.33	1.33±1.22	2.02±0.11	**0.001**
**Biochemical analysis**	182.77	225.06 ±16.83	248.30 ±16.83	0.211
**Microbial level**	7.45	7.33 ±0.17	7.05 ±0.19	0.188

Values in bold indicate significant difference.

Intergroup comparison at one-month follow-up (15 days and one month) was done using ANCOVA after adjusting the respective baseline scores. There were no differences seen in the mean values for Pi (p = 0.791), Gi (p = 0.594), PPD (p = 0.134), protein thiol levels (p = 0.211), and microbial levels (p = 0.188) between the two groups ( Table 4). No harms were reported by any patient.

## Discussion

The present study is the first clinical trial to evaluate the role of goji berry mouthwash in managing periodontitis. The study assessed the efficacy of goji berry mouthwash compared to 0.2% chlorhexidine mouthwash in controlling the gingival inflammation, plaque formation, BOP, PPD, CAL, microbial profile, and antioxidant levels (protein thiols) in the saliva. We found that the mean reduction in Gi, Pi, BOP, and PPD with goji berry mouthwash was comparable to chlorhexidine mouthwash. The reduction in CAL was better for chlorhexidine mouthwash compared to goji berry mouthwash. However, we did not find any statistically significant difference between the goji berry mouth and chlorhexidine mouthwash for Gi, Pi, BOP, and PPD at the end of one month. We compared the goji berry mouthwash to chlorhexidine since chlorhexidine is considered the gold standard mouthwash for managing patients with periodontitis. However in the current times when oral bacteria are becoming resistant to chlorhexidine, there is a need to look for viable alternatives to chlorhexidine for managing periodontitis. ^51^^–^^54^ Additionally, it is known that since prolonged use of chlorhexidine is not advised for more than one month and oral bacteria may develop resiatnce within 10 days, this preliminary study was done with only a small pilot sample size with recall visits for one months. ^55^^,^^56^ Prolonged and continuous use of chlorhexidine is contraindicated to prevent the onset of various side effects such as alterations in taste, staining, and increased calculus, alternatives to chlorhexidine become important. Costa et al. (2017) in a systematic review and meta-analysis also concluded that adjunctive use of chlorhexidine with SRP provides only a minor PPD reduction compared to SRP alone. Therefore, clinicians should consider the small additional gain in PPD reduction, negligible effect on CAL, and potential for adverse effects when prescribing chlorhexidine to their patients. ^52^^–^^54^

Based on our findings, goji berry mouthwash increased the protein thiol levels in saliva, unlike chlorhexidine. This is a positive finding that goji berry would be advantageous compared to chlorhexidine in increasing the antioxidant levels in saliva in patients with periodontal disease. Since periodontitis is linked with increased oxidative stress and free radical-mediated tissue injury, the use of compounds with antioxidant properties like goji berry would be beneficial to control to reduce oxidative stress and control the periodontal inflammation. The increase in the antioxidant levels in saliva can be attributed to the presence of *Lycium barbarum* polysaccharides (LBPs), catechin, epicatechin, quercetin, chlorogenic acid, citric acid, coumaric acid, scopoletin, linoleic acid, kaempferol, and coumaric acid in goji berry. ^51^^–^^58^ The flavonoids in goji berry are proven to have good antimicrobial effects and this justifies the reduction in the Pi and control of gingival inflammation with goji berry mouthwash. ^59^ The reduction in gingival inflammation can also be attributed to the ability of goji berry to inhibit proinflammatory cytokines, radical scavenging activities, and interactions with other antioxidants. Previous studies have shown that goji berries can improve fibroblast healing and increase collagen formation, which could be beneficial for the healing of periodontal pockets and improving clinical attachment levels. ^41^^,^^60^^–^^63^

Another study by Sanghavi et al. (2022) also noted that goji berry has good antimicrobial, anti-adhesion, and anti-biofilm properties against periodontal pathogens ( *P. gingivalis, Aggregatibacter actinomycetemcomitans, Fusobacterium nucleatum, Prevotella intermedia, Tanerella forsythia*). Goji berry extract could inhibit the growth of all these key periodontal pathogens, however, the zone of inhibition was less when compared to doxycycline and chlorhexidine. The anti-adhesion properties of goji berry (96%) were comparable compared to chlorhexidine (96.3%). However, the anti-biofilm activity of chlorhexidine (96%) was found to be slightly better than that of goji berry extract (91.6%). ^49^

Studies by Du et al. (2014) and Ren et al. (2012) also found that goji berry supplementation enhances the maturation and increases the recruitment of neutrophils and monocytes to sites of infection. ^64^^,^^65^ This ability of goji berry to modulate the immune cells also proves its ability to control inflammation, which would be beneficial in lowering the microbial load and controlling periodontal inflammation. Lai et al. (2022) conducted an *in-vitro* and animal study to assess the alkaline phosphatase activity and osteogenic potential of human periodontal ligament stem cells upon treatment with goji berry and found that goji berry can enhance the proliferation and migration of stem cells. This could facilitate superior healing and control of gingival inflammation. The study also noted a reduction in alveolar bone resorption. ^66^

Hence based on our results and existing evidence, it can be stated that goji berry goji berry mouthwash has good antimicrobial and antioxidants for managing periodontitis and can used as an adjunct to SRP for managing periodontitis. However, one should also note that the present study evaluated the role of goji berry for only a short recall time of one month and prolonged exposure to chlorhexidine mouthwash was avoided to prevent side effects. One should also note that although the patients were blinded by using coded amber-colored bottles to mask the color of the mouthwash, the taste of each mouthwash was different. The calibration of mouthwash has not been done. The study was conducted on patients with mild to moderate periodontitis. Therefore, future studies should assess the efficacy of goji berry mouthwash for managing periodontitis with no longer follow-up. Future studies should also evaluate the effect of goji berry on specific periodontal pathogens or local and systemic inflammatory markers for periodontitis.

## Conclusion

Goji berry mouthwash along with SRP was effective in reducing the Gi Pi, BOP, and PPD in patients with periodontitis. No statistically significant difference was noted between the use of goji berry mouthwash and chlorhexidine mouthwash. However, goji berry mouthwash was more effective than chlorhexidine in increasing the antioxidant levels (protein thiol) in saliva.

### Ethical statement

The study was conducted after receiving ethical approval from the Kasturba Medical College and Kasturba Hospital Ethic Committee with IEC no: 117/2019. The trial has been registered at the ‘Clinical Trial Registry (CTRI/2019/05/019042)’.

## Data availability

Figshare: Data on study titled: Lycium barbarum (Goji berry) mouthwash is a viable alternative to 0.2% chlorhexidine gluconate for managing chronic periodontitis: a randomized clinical trial; doi: 10.6084/m9.figshare.21834939. ^67^

This project contains the following data:

• CONSORT checklist
• CONSORT flow diagram

Data are available under the terms of the Creative Commons Attribution 4.0 International license (CC-BY 4.0).

The datasets related to our study is also available with the corresponding author and can be shared on reasonable request via email to aditi.chopra@manipal.edu.
