# Supplementary material for: Efficacy of Lycium barbarum (Goji berry) mouthwash for managing periodontitis: a randomized clinical trial
Source: F1000Res. 2024 Nov 26;12:302. Originally published 2023 Mar 20. [Version 4] doi: 10.12688/f1000research.129891.4 (PMC11617829; doi:10.12688/f1000research.129891.4)
Supplement: Peer review history

# Reviewer report: Reviewer response for version 4

**DOI:** 10.5256/f1000research.174704.r356385

**Published:** 2025-03-06

**Authors:** Dr. Namitha P Kamath

**Affiliations:** 1 AB Shetty Memorial Institute of Dental Sciences, Mangaluru, Karnataka, India

The study and methodology have been well designed, mainly the figures 1 and 2 explain it well. However, the microbial analysis during the stability test trial of the mouthwash has been shown in the figure 1 but not explained in the methodology text.

 Sample size can be increased to evaluate the effects of the mouthwash better, so that or before, it can be commercially used as an alternative to the Chlorhexidine mouthwash.

**Methods**: The inclusion and exclusion criteria have been well formatted. But as per point 8 of exclusion criteria, even the different types of dentifrice used by each participant varies. This can also add to the bias in the results. It has not been mentioned whether all participants were given the same dentifrice during the study duration.

**Methods**:

 c. Examination of the clinical periodontal parameters:

 Why were the participants of Goji berry mouthwash instructed to dilute it and the participants of chlorhexidine mouthwash requested to use undiluted mouthwash? 

 During follow up, Substantively of the mouthwash can be tested, if a follow up is included for 7 or 15 days after washout period, i.e. after stopping the use of mouthwash, for both the groups.

 Also, a Placebo group could have been included to compare the results.

**Results **have been explained well.

**Discussion: A certain grammatical errors and repetition of words have been found.**

**In the article: **(Prolonged and continuous use of chlorhexidine is contraindicated to prevent the onset of various side effects such as alterations in taste, staining, and increased calculus, alternatives to chlorhexidine become important.)

**Can be corrected as:** Prolonged and continuous use of chlorhexidine is contraindicated to prevent the onset of various side effects such as alterations in taste, staining, and increased calculus. **Hence **alternatives to chlorhexidine become important.

**In the article**: The increase in the antioxidant levels in saliva can be attributed to the presence of *Lycium barbarum* polysaccharides (LBPs), catechin, epicatechin, quercetin, chlorogenic acid, citric acid, **coumaric acid,** scopoletin, linoleic acid, kaempferol, and **coumaric acid** in goji berry. ^51^^–^^58^

**Coumaric acid has been typed twice.**

**In the article: **The anti-adhesion properties of goji berry (96%) were comparable compared to chlorhexidine (96.3%).

**Can be corrected as: **The anti-adhesion properties of goji berry (96%) were comparable to that of chlorhexidine (96.3%).

**In the article**: Hence based on our results and existing evidence, it can be stated that **goji berry goji berry **mouthwash has good antimicrobial and antioxidants for managing periodontitis

**Goji berry has been typed twice**

**In the article: **Therefore, future studies should assess the efficacy of goji berry mouthwash for managing periodontitis with **no **longer follow-up.

 Is the word **NO**, typed by mistake?

**References**:

 There have been past studies in the MCODS, Manipal, where other Alternatives of Herbal mouthwashes like Triphala, Aloe vera, Tea Tree oil, etc. have been used in comparison to Chlorhexidine mouthwash.

Is the work clearly and accurately presented and does it cite the current literature?

Yes

If applicable, is the statistical analysis and its interpretation appropriate?

I cannot comment. A qualified statistician is required.

Are all the source data underlying the results available to ensure full reproducibility?

Yes

Is the study design appropriate and is the work technically sound?

Yes

Are the conclusions drawn adequately supported by the results?

Yes

Are sufficient details of methods and analysis provided to allow replication by others?

Yes

Reviewer Expertise:

Prevention of Caries, in Pediatric age group, so that quality of life is improved during the earliest part of life itself.

I confirm that I have read this submission and believe that I have an appropriate level of expertise to confirm that it is of an acceptable scientific standard, however I have significant reservations, as outlined above.

# Reviewer report: Reviewer response for version 4

**DOI:** 10.5256/f1000research.174704.r351776

**Published:** 2025-01-07

**Authors:** Gaetano Isola

**Affiliations:** 1 University of Catania, Catania, Italy

In the manuscript entitled: "Efficacy of Lycium barbarum (Goji berry) mouthwash for managing periodontitis: a randomized clinical trial" the authors aimed to analyze the efficacy of goji berry mouthwash compared to chlorhexidine mouthwash for managing periodontitis.

 The authors concluded that Goji berry mouthwash along with SRP reduced the Gi, Pi, BOP, and PPD in patients with periodontitis. However, no statistically significant difference was noted between the use of goji berry and chlorhexidine mouthwash. Goji berry mouthwash was more effective than chlorhexidine in increasing the antioxidant levels in saliva.

**Major comments:**

 In general, the idea and innovation of this study regards the analysis of periodontitis and related effects and mediators is interesting and novel because the role these aspects in medicine are validated but further studies on this topic could be an innovative issue in this field could be open a creative matter of debate in literature by adding new information.

 The study was well conducted by the authors; However, there are some concerns to revise that are described below.

 The introduction section resumes the existing knowledge regarding the important factor linked with periodontal treatment and related mediators.

 However, as the importance of the topic, the reviewer recommends to update the literature through read, discuss some recent interesting articles, that helps the authors to better introduce and discuss the role of periodontal treatment approaches and related mediators that could impact the development of periodontitis evolution especially about CVD risk 1) Polizzi A.et.al 2024 (Ref 1) 2.) Isola G.et.al 2024 (Ref 2)

 The authors should be better specified, at the end of the introduction section, the rationale of the study and the aim of the study. In the central section, should better clarify inclusions and exclusions criteria of the selected sample.

 Please better state the results obtained in the abstract.

 The discussion section appears well organized with the relevant paper that support the conclusions, even if the authors should better discuss the relationship regarding by periodontal treatment approaches that could improve the quality of life in periodontitis patients. The conclusion should reinforce in light of the discussions.

 In conclusion, I am sure that the authors are fine clinicians who achieve very nice results with their adopted protocol.

**Minor Comments:**

 Abstract: Better formulate the abstract section by better describing the aim of the study

 Introduction: Please refer to major comments

 Discussion: Please add a specific sentence that clarifies the results obtained in the first part of the discussion

Is the work clearly and accurately presented and does it cite the current literature?

No

If applicable, is the statistical analysis and its interpretation appropriate?

Partly

Are all the source data underlying the results available to ensure full reproducibility?

Partly

Is the study design appropriate and is the work technically sound?

Yes

Are the conclusions drawn adequately supported by the results?

Partly

Are sufficient details of methods and analysis provided to allow replication by others?

Partly

Reviewer Expertise:

dentistry

I confirm that I have read this submission and believe that I have an appropriate level of expertise to confirm that it is of an acceptable scientific standard, however I have significant reservations, as outlined above.

## References

[1] : Impact of nonsurgical periodontal treatment on arterial stiffness outcomes related to endothelial dysfunction: A systematic review and meta-analysis. *J Periodontol* .2024; 10.1002/JPER.24-0422 10.1002/JPER.24-0422 39549247 PMC12062727

[2] : Effect of quadrantwise versus full-mouth subgingival instrumentation on clinical and microbiological parameters in periodontitis patients: A randomized clinical trial. *J Periodontal Res* .2024;59(4) : 10.1111/jre.13279 647-656 10.1111/jre.13279 38666373

# Reviewer report: Reviewer response for version 4

**DOI:** 10.5256/f1000research.174704.r343647

**Published:** 2024-12-04

**Authors:** Paulo José Lima Juiz

**Affiliations:** 1 Federal University of Recôncavo da Bahia, Feira de Santana, Brazil

The authors provided a comprehensive review of the work, which  enhances the clarity and understanding of the results. This thoughtful approach helps to highlight the key findings.

Is the work clearly and accurately presented and does it cite the current literature?

Partly

If applicable, is the statistical analysis and its interpretation appropriate?

I cannot comment. A qualified statistician is required.

Are all the source data underlying the results available to ensure full reproducibility?

No

Is the study design appropriate and is the work technically sound?

No

Are the conclusions drawn adequately supported by the results?

No

Are sufficient details of methods and analysis provided to allow replication by others?

No

Reviewer Expertise:

Biotechnology

I confirm that I have read this submission and believe that I have an appropriate level of expertise to confirm that it is of an acceptable scientific standard.

# Reviewer report: Reviewer response for version 3

**DOI:** 10.5256/f1000research.168737.r324931

**Published:** 2024-10-10

**Authors:** Paulo José Lima Juiz

**Affiliations:** 1 Federal University of Recôncavo da Bahia, Feira de Santana, Brazil

The article is pertinent and highlights the extract's therapeutic potential. However, the sample size was insufficient to conclusively support its use in periodontal therapy. Furthermore, to validate the results, it is essential to assess groups that only underwent scaling and root planning (SRP) and groups treated with the vehicle used in the formulation without the extract. Therefore, I recommend further research to strengthen the findings before presenting the article with more substantial data in the future.

 Below are some compelling observations:

**1) Written in the text:** Periodontitis is considered the 6th most common disease, affecting approximately 20–50% of the adult population worldwide.

 Please ensure that the epidemiological data specified is from 2017 and provide more recent data, if available.

 2) **Rewrite the sentence:** It is primarily caused by the **IMMUNE RESPONDE DUE TO THE** interaction of the gingival and periodontal tissues with the microorganisms in the oral biofilm

 3) Regarding Figure 1, should it be labeled as MMT instead of MTT?

 4) **Written in the text:** …mixed in 1.15 of 100% alcohol and then homogenized using 15% glycerol.

 Kindly specify the alcohol concentration in Methods. Did the authors use methanol and ethanol?

 5) Without the extract in the formulation, could you confirm if this formulation is considered active?

 A concentration of 50 ug/ml was mixed in 1.15 of 100% alcohol and then homogenized using 15% glycerol. To this around 15% propylene glycol and 1% Tween solution was added to formulate the mouthwash. 0.1% menthol was added to adjust the taste of the mouthwash. The volume was adjusted to 100% by using distilled water. 100% alcohol was added to serve both as a preservative and a dissolvent.

 6) Shouldn't Table 1 be classified as a result?

 7) Please provide the reference for the classification presented.

**Written in the text:** Ninety subjects aged 20 to 50 years (both male/females) visiting the outpatient department were screened for the presence of localized/generalized periodontitis according to the 2017 classification of periodontal disease

 8) The description of the microbiological method appears to be insufficient. Can you furnish details such as the reference, growing conditions, anaerobiosis, and the culture media used?

**Written in the text:** The plaque from the subgingival region was collected from the pocket with the maximum probing depth in each quadrant using a sterile site-specific Gracey curette. After collection, the curette was gently submerged in a reduced transport medium (Thioglycolate bath) for testing the total colony Forming Units (CFU). The number of colonies was calculated on the plated blood agar and then converted to CFU/mL using the following formula: CFU/mL = (no. of colonies × dilution factor)/volume of the culture plate.

 9) Is it a thioglycolate bath or broth?

 10) Figure 1, which depicts a schematic representation of the study design, notes that MTT was conducted between tests, yet the method and results were not described. Was the test cytotoxic?

 11) BOP needed to be delineated in the results text.

 12) **Written in the text:** Since goji berry mouthwash showed comparable results to chlorhexidine, it can be used along with SRP for managing periodontitis **, especially in the current times when oral bacteria are becoming resistant to chlorhexidine**

 After one month, does the bacteria become resistant?

 13) I did not observe the microbiological results in RESULTS and DISCUSSION.

 14) Can we confirm the veracity of the following statement?

**Written in the text:** It can be stated that goji berry has a promising role in managing periodontitis.

Is the work clearly and accurately presented and does it cite the current literature?

Partly

If applicable, is the statistical analysis and its interpretation appropriate?

I cannot comment. A qualified statistician is required.

Are all the source data underlying the results available to ensure full reproducibility?

No

Is the study design appropriate and is the work technically sound?

No

Are the conclusions drawn adequately supported by the results?

No

Are sufficient details of methods and analysis provided to allow replication by others?

No

Reviewer Expertise:

Biotechnology

I confirm that I have read this submission and believe that I have an appropriate level of expertise to state that I do not consider it to be of an acceptable scientific standard, for reasons outlined above.

# Response

**Published:** 2024-11-11

**Authors:** Aditi Chopra

**Affiliations:** Periodontology, Manipal College of Dental Sciences, Manipal, Manipal, India

Firstly, we would like to thank the reviewers for their time and provide valuable insights for improving our work. We are truly thankful for the same. We have revised the manuscript as suggested (in track change mode). However, we are truly grateful for the valuable suggestion and will plan for further research based on the valuable recommendations. We agree with the reviewers and their suggestions and responses to each comment are as follows. The changes in the manuscript are highlighted in track change mode. We would like to humbly request to note that the study was designed to test the effect of a new mouthwash. Hence it was conducted as a pilot randomized study with a small sample size to check the effect of goji berry mouthwash on patients with periodontitis for the first time.

**Comment 1:** Written in the text: Periodontitis is considered the 6th most common disease, affecting approximately 20–50% of the adult population worldwide. Please ensure that the epidemiological data specified is from 2017 and provide more recent data, if available.

**Response: **We have revised the data and added recent data on the prevalence of periodontitis as follows from articles from Nascimento et al (2024): “In 2021, over 1 billion people were affected by severe periodontitis, with a global age-standardized prevalence of 12.50%. South Asia had the highest prevalence rate, at 17.57%.”

*Reference: Nascimento GG, Alves-Costa S, Romandini M. Burden of severe periodontitis and edentulism in 2021, with projections up to 2050: The Global Burden of Disease 2021 study. J Periodontal Res. 2024 Oct;59(5):823-867. doi: 10.1111/jre.13337.*

**Comment 2**: Rewrite the sentence: It is primarily caused by the Immune response due to the interaction of the gingival and periodontal tissues with the microorganisms in the oral biofilm

**Response:** We thank the reviewers for the valuable insights. We have revised the sentence as follows: “It is primarily caused by the interaction of the oral microorganisms with the host (soft tissues surrounding the teeth. This host-microbial interaction triggers a massive influx of various pro-inflammatory mediators, microbial by-products, proteolytic enzymes, and free reactive oxygen species (ROS) leading to periodontal tissue destruction and inflammation”

**Comment 3:** Regarding Figure 1, should it be labeled as MMT instead of MTT?

**Response:** We have revised the figure as Figure 1 and corrected the Typo error.

**Comment 4:** Written in the text: …mixed in 1.15 of 100% alcohol and then homogenized using 15% glycerol. Kindly specify the alcohol concentration in Methods. Did the authors use methanol and ethanol?

**Response:** We thank the reviewer for pointing out this inadequacy in content. We have used ethanol, and the same has been added to the text.

**Comment 5: **Without the extract in the formulation, could you confirm if this formulation is considered active? A concentration of 50 ug/ml was mixed in 1.15 of 100% alcohol and then homogenized using 15% glycerol. To this around 15% propylene glycol and 1% Tween solution was added to formulate the mouthwash. 0.1% menthol was added to adjust the taste of the mouthwash. The volume was adjusted to 100% by using distilled water. 100% alcohol was added to serve both as a preservative and a dissolvent.

**Response:** The active ingredient is an extract at a concentration of 50 µg/ml. Since alcohol is present at 1.15%, it is not an active antimicrobial agent. Alcohol must be present in the final product at a concentration of around 15-20% for it to contribute as an active antimicrobial ingredient. Anything below this level will primarily act as a preservative or solvent aid. The other components, such as glycerol, propylene glycol, and tween, are inactive and act as a solvent and stabilizer. Menthol is considered inactive in terms of antimicrobial activity at this concentration and is used to provide taste and a cooling sensation.

*Ref: Jones, C. G. (1997). Chlorhexidine: Is it still the gold standard? Periodontology 2000, 15(1), 55-62.*

**Comment 6:** Shouldn't Table 1 be classified as a result?

**Response:** We thank the reviewer for their valuable insight, we have revised the section and now included it in the result section as suggested.

**Comment 7:** Please provide the reference for the classification presented. Written in the text: Ninety subjects aged 20 to 50 years (both male/females) visiting the outpatient department were screened for the presence of localized/generalized periodontitis according to the 2017 classification of periodontal disease.

**Response:** We thank the reviewer for their valuable insights. We have added the following reference for the classification system for periodontal disease.

*Papapanou PN, Sanz M, Buduneli N, Dietrich T, Feres M, Fine DH, Flemmig TF, Garcia R, Giannobile WV, Graziani F, Greenwell H, Herrera D, Kao RT, Kebschull M, Kinane DF, Kirkwood KL, Kocher T, Kornman KS, Kumar PS, Loos BG, Machtei E, Meng H, Mombelli A, Needleman I, Offenbacher S, Seymour GJ, Teles R, Tonetti MS. Periodontitis: Consensus report of workgroup 2 of the 2017 World Workshop on the Classification of Periodontal and Peri-Implant Diseases and Conditions. J Periodontol. 2018 Jun;89 Suppl 1:S173-S182. doi: 10.1002/JPER.17-0721.*

**Comment 8:** The description of the microbiological method appears to be insufficient. Can you furnish details such as the reference, growing conditions, anaerobiosis, and the culture media used? Written in the text: The plaque from the subgingival region was collected from the pocket with the maximum probing depth in each quadrant using a sterile site-specific Gracey curette. After collection, the curette was gently submerged in a reduced transport medium (Thioglycolate bath) for testing the total colony Forming Units (CFU). The number of colonies was calculated on the plated blood agar and then converted to CFU/mL using the following formula: CFU/mL = (no. of colonies × dilution factor)/volume of the culture plate.

**Response:** We thank the reviewer for the insight. We have revised the text in the document as follows:

 “The plaque from the subgingival region was collected from the pocket with the greatest probing depth in each quadrant using a sterile, site-specific Gracey curette. Following collection, the curette was promptly immersed in a reduced transport medium (Thioglycolate broth supplemented with hemin and vitamin K) for microbiological analysis. Each sample underwent vortexing, and quantitative cultures were performed across various dilutions (1:10, 1:100, 1:1000, and 1:10,000). A volume of 10 µl from each dilution was plated onto pre-reduced 5% sheep blood agar (BD, Becton Dickinson, Heidelberg, Germany), with plates incubated at 37°C for 48 hours in an anaerobic chamber (Whitley A35 Anaerobic workstation, Don Whitley Scientific, Shipley, UK). The colony-forming units were analyzed for each sample using the following formula: CFU/mL = (number of colonies × dilution factor)/volume of the culture plate.”

**Comment 9:** Is it a thioglycolate bath or broth?

**Response:** We apologize for the Typo error, We have changed the word to “ broth”.

**Comment 10:** Figure 1, which depicts a schematic representation of the study design, notes that MTT was conducted between tests, yet the method and results were not described. Was the test cytotoxic?

**Response:** We have modified the figure as suggested.

**Comment 11:** BOP needed to be delineated in the results text.

**Response:** We have revised the results section and added data on BOP as follows: “The percentage of sites with BOP reduced from 84.8± 0.32 at baseline in the test group to 09.0± 0.32 at the end of six months. These results were comparable to the control group with no statistically significant difference between the test and control”.

**Comment 12:** Written in the text: Since goji berry mouthwash showed comparable results to chlorhexidine, it can be used along with SRP for managing periodontitis, especially in the current times when oral bacteria are becoming resistant to chlorhexidine. After one month, does the bacteria become resistant?

**Response:** Studies have found that oral bacteria can become resistant to chlorhexidine and time and amount of resistance develop depending upon the concentration and duration of chlorhexidine used, and the nature of the microorganism. In an article by Fruh et al 2022, the authors investigated the adaptation of oral bacteria to chlorhexidine digluconate (CHX) and potential cross-adaptation to antibiotics after repeated exposure of supragingival plaque samples to subinhibitory concentrations of CHX. Plaque samples from six healthy donors were passaged for 10 days in subinhibitory concentrations of CHX while passaging of plaque samples without CHX served as a control. Subsequently, the minimum inhibitory concentrations (MIC) of these isolates toward CHX were determined using a broth-microdilution method, and phenotypic antibiotic resistance was evaluated using the epsilometer test. Repeated exposure of supragingival plaque samples to subinhibitory concentrations of CHX led to the selection of oral bacteria with 2-fold up to 4-fold increased MICs toward CHX. Furthermore, these isolates showed up to 12-fold increased MICs towards some antibiotics such as erythromycin and clindamycin. Conversely, biofilm-forming capacity was decreased. Wang et al. (2017a) evaluated changes in MIC in eight common oral bacterial species over 10 passages of CHX-challenge and re-growth in CHX-free medium and reported adaptation in *Streptococcus gordonii, E. faecalis, Fusobacterium nucleatum*, and *P. gingivalis*. Further analysis of the adapted *S. gordonii* strain showed delayed growth with prolonged log-phase and decelerated growth rate as compared to its parental strain indicating reduced metabolic activity that may be responsible for the reduced susceptibility toward CHX (Wang et al., 2017a).  

*Ref: Früh R, Anderson A, Cieplik F, Hellwig E, Wittmer A, Vach K, Al-Ahmad A. Antibiotic Resistance of Selected Bacteria after Treatment of the Supragingival Biofilm with Subinhibitory Chlorhexidine Concentrations. Antibiotics (Basel). 2022 Oct 17;11(10):1420. doi: 10.3390/antibiotics11101420. PMID: 36290078; PMCID: PMC9598507.*

**Comment 13:** I did not observe the microbiological results in RESULTS and DISCUSSION.

**Response:** We thank the reviewers for their valuable insights. We have now added the microbiological section to the results section in both the abstract and text.

**Comment 14:** Can we confirm the veracity of the following statement? Written in the text: It can be stated that goji berry has a promising role in managing periodontitis.

**Response:** We have modified the sentence as follows: It can be stated that goji berry can be used as an adjunct to SRP for managing periodontitis. Based on the previous pieces of evidence (in-vitro and in-vivo studies) that confirm the antioxidant and antimicrobial properties of goji berry and previously published reports on the efficacy of oral bacteria on periodontal pathogens, we can conclude that goji berry is a good source of antimicrobial and antioxidants for managing periodontitis.

# Reviewer report: Reviewer response for version 3

**DOI:** 10.5256/f1000research.168737.r299638

**Published:** 2024-07-17

**Authors:** Francisco Wilker Mustafa Gomes Muniz

**Affiliations:** 1 Graduate Program in Dentistry, Department of Periodontology, Universidade Federal de Pelotas, Pelotas, State of Rio Grande do Sul, Brazil

No further comments.

Is the work clearly and accurately presented and does it cite the current literature?

Partly

If applicable, is the statistical analysis and its interpretation appropriate?

Partly

Are all the source data underlying the results available to ensure full reproducibility?

No

Is the study design appropriate and is the work technically sound?

Partly

Are the conclusions drawn adequately supported by the results?

No

Are sufficient details of methods and analysis provided to allow replication by others?

No

Reviewer Expertise:

Periodontology

I confirm that I have read this submission and believe that I have an appropriate level of expertise to confirm that it is of an acceptable scientific standard.

# Reviewer report: Reviewer response for version 2

**DOI:** 10.5256/f1000research.158544.r223446

**Published:** 2023-12-14

**Authors:** Francisco Wilker Mustafa Gomes Muniz

**Affiliations:** 1 Graduate Program in Dentistry, Department of Periodontology, Universidade Federal de Pelotas, Pelotas, State of Rio Grande do Sul, Brazil

This is the second round of peer review of this referee. Overall, the quality of the manuscript increased significantly, and authors have answered most of my queries properly. However, some issues still remain.

**Methods**

- Remove the following sentence: “1. Individuals with severe periodontitis (localized or generalized stage III and stage IV) were excluded.” Authors have included only individuals with periodontitis stage I and II. Therefore, it is not possible to exclude stage III and IV.
- For periodontal assessment, authors wrote the following: “The deepest pocket depth at each surface was recorded. The average of each surface was considered as the reading for that tooth.” It is clear now that six sites were measured, but only four were record. However, was is the deepest one or a mean of both interproximal surfaces?
- It is still unclear what commercial brand, if any, was used for chlorhexidine.
- Chlorhexidine was diluted in water. This is not appropriate and must be discussed. In fact, a very lower concentration of the product was achieved by this protocol.
- The lack of calibration process is worrisome and must be discussed.

**Discussion**

- Remove the following sentence: “In such situations, the use of herbal mouthwash like goji berry would be a viable alternative. ^51^” Firstly, the very low additional effect of chlorhexidine is not a justification for the use of herbal mouthwash. In addition, reference 51 do not advise for the use of goji berry.
- Italicize “in vitro” in the whole manuscript, as previously requested.
- Correct the following sentence to: “The study was conducted on patients with mild to moderate  periodontitis. Therefore, future studies should assess the efficacy of goji berry mouthwash on severe cases of periodontitis with longer follow-up.”

Is the work clearly and accurately presented and does it cite the current literature?

Partly

If applicable, is the statistical analysis and its interpretation appropriate?

Partly

Are all the source data underlying the results available to ensure full reproducibility?

No

Is the study design appropriate and is the work technically sound?

Partly

Are the conclusions drawn adequately supported by the results?

No

Are sufficient details of methods and analysis provided to allow replication by others?

No

Reviewer Expertise:

Periodontology

I confirm that I have read this submission and believe that I have an appropriate level of expertise to confirm that it is of an acceptable scientific standard, however I have significant reservations, as outlined above.

# Response

**Published:** 2024-07-02

**Authors:** Aditi Chopra

**Affiliations:** Periodontology, Manipal College of Dental Sciences, Manipal, Manipal, India

Firstly, we would like to thank the reviewers for their time and provide valuable insights for improving our work. We are truly thankful for the same. We have revised the manuscript as suggested (in track change mode). The responses to each comment are as follows:

 Comment 1: Remove the following sentence: “1. Individuals with severe periodontitis (localized or generalized stage III and stage IV) were excluded.” Authors have included only individuals with periodontitis stage I and II. Therefore, it is not possible to exclude stages III and IV.

 Response: We have removed the line from the exclusion criteria as suggested

 Comment 2: For periodontal assessment, authors wrote the following: “The deepest pocket depth at each surface was recorded. The average of each surface was considered as the reading for that tooth.” It is clear now that six sites were measured, but only four were recorded. However, was it the deepest one or a mean of both interproximal surfaces?

 Response: We have revised the paper and added the line that means the interdental area was recorded to take the overall measurement for the interdental site.

 Comment 3: It is still unclear what commercial brand, if any, was used for chlorhexidine.

 Response: We added the name of the brand used for chlorhexidine

 Comments 4: Chlorhexidine was diluted in water. This is not appropriate and must be discussed. A very low concentration of the product was achieved by this protocol.

 Response: We have revised the ambiguity in the paper. We would like to highlight that Hexidine mouthwash (brand name was given to all the patients) and the patients were asked not to dilute the mouthwash. For the goji berry mouthwash, 10 ml of water was used to dilute.

 Comment 5: The lack of a calibration process is worrisome and must be discussed.

 Response: we have discussed the lack of calibration process in the limitations of the study in the discussion.

 Comment 6: Remove the following sentence: “In such situations, the use of herbal mouthwash like goji berry would be a viable alternative.51” Firstly, chlorhexidine's very low additional effect is not a justification for the use of herbal mouthwash. In addition, reference 51 does not advise for the use of goji berries.

 Response: We have removed the abovementioned sentence as suggested.

 Comment 7: Italicize “in vitro” in the whole manuscript, as previously requested.

 Response: We have Italicized the word ‘in vitro’ throughout the manuscript.

 Comment 8: Correct the following sentence to: “The study was conducted on patients with mild to moderate periodontitis. Therefore, future studies should assess the efficacy of goji berry mouthwash on severe cases of periodontitis with longer follow-up.”

 Response: We have corrected the following lines as suggested.

# Reviewer report: Reviewer response for version 1

**DOI:** 10.5256/f1000research.142608.r205506

**Published:** 2023-10-09

**Authors:** Francisco Wilker Mustafa Gomes Muniz

**Affiliations:** 1 Graduate Program in Dentistry, Department of Periodontology, Universidade Federal de Pelotas, Pelotas, State of Rio Grande do Sul, Brazil

This is the first round of peer review of this referee. Several queries were raised as stated below:

**Overall**

- A very short follow-up period was followed for the chosen outcomes, such as BOP, CAL and PPD. A longer follow-up period, to detect any important change, is necessary. The antiplaque and antigingivitis efficacy may be important to the literature, though.
- Throughout the manuscript the term “periodontal disease” is used, maybe, referring to periodontitis.  The term periodontal disease is very broad, and do not mean only periodontitis. Authors must revise it.

**Title**

- Chlorhexidine is an excellent mouthwash to control biofilm and gingival inflammation. However, literature does not state that this mouthwash is useful for the treatment of periodontitis. Other treatments are more efficacious for this periodontal disease. Therefore, title must be revised, as it may be misleading in its current format. Please see Sanz, M., Herrera, D., Kebschull, M., Chapple, I., Jepsen, S., Berglundh, T., ... & Wennström, J. (2020). Treatment of stage I–III periodontitis—The EFP S3 level clinical practice guideline. Journal of clinical periodontology, 47, 4-60.
- Moreover, the term “chronic periodontitis” should not be used since 2018. Please see Caton, J. G., Armitage, G., Berglundh, T., Chapple, I. L., Jepsen, S., Kornman, K. S., ... & Tonetti, M. S. (2018). A new classification scheme for periodontal and peri‐implant diseases and conditions–Introduction and key changes from the 1999 classification. Journal of periodontology, 89, S1-S8. Prefer the term “periodontitis” whenever appropriate, and this must be applied to all manuscript.
- Additionally, lower gain of CAL was detected in the test group. Title must not state that similar results were detected between groups.

**Abstract**

- Authors wrote: “A statistically significant difference was noted in the Pi (P-value = 0.791), Gi (P-value= 0.594), PPD (with P-value= 0.134), and microbial levels (P-value = 0.188) in both groups from the baseline.” However, none of the showed p-values mean statistical significance. The sentence is not understandable. Does the comparison mean within or between comparisons?
- Conclusion may also be misleading. *L. barbarum* mouthwash presented similar clinical efficacy, when compared to chlorhexidine. The SRP performed may be the main reason to the so-called “effective in managing periodontal disease”. Please provide a interpretation aligned with your aims.

**Introduction**

- The term “exaggerate” in the first paragraph should be revised, as may be not be the most appropriate term in an epidemiological point of view. In addition, authors have cited both risk factors and indicator factors associated with periodontitis.
- Reference is necessary for the following sentence: “However, mechanical debridement is technically demanding, as many times removal of hard and soft tissue deposits from pockets greater than five mm becomes challenging.” In well-trained clinicians, this may not be a problem.
- Provide citation for the following sentences:
  - “Recently, goji berry, commonly known as Wolfberry, Himalayan goji, or Tibetan goji, has gained a lot of popularity due to its strong antioxidant and anti-inflammatory properties.”
  - “The stems and berries of goji berry plant was effective against many various Gram-negative bacteria and Gram-positive bacteria.”
- Italicize the word “in vitro.”
- Reference #47 may not be necessary in the following sentence: “(…) efficacy of *L. barbarum *mouthwash along with SRP for patients with chronic periodontitis compared to chlorhexidine for the first time. ^47^”
- Why salivary levels of protein thiol were assessed in the present study? What is the relevance for the present study. This is not properly explained in this section.

**Objective**

- Pi, Gi, BOP, PPD, and CAL must be explained, as this is the first time their appear in the text.

**Methods**

- Figure 1 do not represent the flowchart of the study, and do not follow what CONSORT recommends. Figure 1 is illustrative and important, but the sentence must be revised.
- Figure 2, in fact, must be placed in the Results section, as recommended by CONSORT.
- Do not use Stage 1, use “stage I.” Provide reference for this inclusion criterion
- Do not mistake exclusion criteria as the opposite of inclusion criteria. For instance, if only generalized periodontitis was included. It is not necessary to exclude those with localized periodontitis.
- What is “chlorhexidineg”?
- Revise the following sentence: “Participants currently using anti-inflammatory, antibiotics, or analgesics in the last six months.” It seems that patients that used antibiotics in the last six months were included.
- Revise exclusion criteria #10, as the sentence is in the future.
- Sample size calculation is not properly described. Readers to not know the necessary number of patients to detect important measures in the primary outcome. For instance, the effect size of 0.7 is referring to what outcome?
- Why allocation stated uneven (30:27) at baseline (Figure 2), as a 1:1 allocation ratio was established in the Methods?
- In fact, allocation concealment is not properly explained.
- Both mouthwashes may be very distinct in the general characteristics, such as taste, color and visual aspects. Therefore, this referee does not understand the following sentence: “The participants were also blinded about their group.”
- It is well-established that the best periodontal examination must be performed in six sites per tooth. It is not clear why authors have performed it only in four.
- Further information regarding chlorhexidine is necessary. Was it manufactured by the authors? What percentage was used? Provide details.
-  Chlorhexidine, such as those commercially available, must not be diluted in water. The lower efficacy of chlorhexidine must not be ruled out in the present study.
- It is not clear for how long (who many seconds) volunteers should rinse.
- Calibration, if any, is not reported.

**Results **

- In Table 3, why p-values for the comparison between groups are reported only for baseline?
- The following sentence should be better placed in the Methods: “(…) repeated measures of ANOVA with Greenhouse Geisser correction’ followed by a ‘post-hoc analysis with Bonferroni adjustment’.”
- Figure 3 and Table 3 are repetitive results. Both of them are not necessary.
- Authors wrote: “One patient reported bitter taste of the mouthwash and discontinued the mouthwash.” What mouthwash?

**Discussion**

- Effectiveness was not tested. Do not mistake it with efficacy.
- Only mild periodontitis patients were included in the present study. Do not generalize it. This type of patient is not discussed.
- The following sentences are not correct: “Based on the results, it was noted that goji berry mouthwash can control the gingival inflammation, BOP, and plaque formation at 15 days following SRP with statistically significant difference compared to chlorhexidine group. A significant in the Gi, Pi, BOP, PPD was noted in participants using goji berry mouthwash.” No differences between groups were detected.
- Again, chlorhexidine is not largely used for the management of periodontitis. In fact, its use as adjuvant to periodontitis treatment is not well-established, and the previously mentioned clinical practice guideline do not recommend it.
- A broader discussion regarding the *L. barbarum* on periodontal tissues is warranted.
- There is no discussion regarding the very short follow-up period.

Is the work clearly and accurately presented and does it cite the current literature?

Partly

If applicable, is the statistical analysis and its interpretation appropriate?

Partly

Are all the source data underlying the results available to ensure full reproducibility?

No

Is the study design appropriate and is the work technically sound?

Partly

Are the conclusions drawn adequately supported by the results?

No

Are sufficient details of methods and analysis provided to allow replication by others?

No

Reviewer Expertise:

Periodontology

I confirm that I have read this submission and believe that I have an appropriate level of expertise to state that I do not consider it to be of an acceptable scientific standard, for reasons outlined above.

## References

[1] : Treatment of stage I-III periodontitis-The EFP S3 level clinical practice guideline. *J Clin Periodontol* .2020;47 Suppl 22(Suppl 22) : 10.1111/jcpe.13290 4-60 10.1111/jcpe.13290 32383274 PMC7891343

[2] : A new classification scheme for periodontal and peri-implant diseases and conditions - Introduction and key changes from the 1999 classification. *J Clin Periodontol* .2018;45 Suppl 20: 10.1111/jcpe.12935 S1-S8 10.1111/jcpe.12935 29926489

# Response

**Published:** 2023-11-04

**Authors:** Aditi Chopra

**Affiliations:** Periodontology, Manipal College of Dental Sciences, Manipal, Manipal, India

Firstly, we would like to thank all the reviewers and the editor for spending their valuable time and providing us with their valuable comments and suggestions. We truly appreciate your guidance in improving our work. We have tried to address the comments and revised the manuscript accordingly. The changes in the manuscript have been highlighted in track change mode in the revised manuscript.

 Reviewer 2

  

- A very short follow-up period was followed for the chosen outcomes, such as BOP, CAL, and PPD. A longer follow-up period, to detect any important change is necessary. The antiplaque and antigingivitis efficacy may be important to the literature, though. Throughout the manuscript the term “periodontal disease” is used, maybe, referring to periodontitis.  The term periodontal disease is very broad, and do not mean only periodontitis. The authors must revise it.

Response: We thank the reviewers for their valuable insight. We agree with the reviewer that a long-term follow-up is necessary, however, due to various side effects of chlorhexidine it is not advised to use for a long time. Additionally, there are studies that have found the development of antimicrobial resistance in periodontal pathogens against Chlorhexidine, hence the use of herbal mouthwashes is constantly being researched. We have also revised the term periodontal disease to periodontitis as suggested. The changes in the manuscript have been highlighted in track change mode in the revised manuscript.

  

- Chlorhexidine is an excellent mouthwash to control biofilm and gingival inflammation. However, literature does not state that this mouthwash is useful for the treatment of periodontitis. Other treatments are more efficacious for this periodontal disease. Therefore, title must be revised, as it may be misleading in its current format. Please see Sanz, M., Herrera, D., Kebschull, M., Chapple, I., Jepsen, S., Berglundh, T., ... & Wennström, J. (2020). Treatment of stage I–III periodontitis—The EFP S3 level clinical practice guideline. Journal of Clinical Periodontology, 47, 4-60. Moreover, the term “chronic periodontitis” should not be used since 2018. Please see Caton, J. G., Armitage, G., Berglundh, T., Chapple, I. L., Jepsen, S., Kornman, K. S., ... & Tonetti, M. S. (2018). A new classification scheme for periodontal and peri‐implant diseases and conditions–Introduction and key changes from the 1999 classification. Journal of periodontology, 89, S1-S8. Prefer the term “periodontitis” whenever appropriate, and this must be applied to all manuscript.

Response: we thank the reviewers for sharing the article. Based on your suggestion we have now revised the title as follows: Efficacy of Lycium barbarum (Goji berry) mouthwash for managing periodontitis: a randomized clinical trial. We have now revised the terminology chronic periodontitis to periodontitis as requested. The changes in the manuscript have been highlighted in track change mode in the revised manuscript.

  

- Additionally, lower gain of CAL was detected in the test group. Title must not state that similar results were detected between groups.

Response: we have now revised the interpretation as requested both in abstract and result/ discussion section. 

 Abstract

- Authors wrote: “A statistically significant difference was noted in the Pi (P-value = 0.791), Gi (P-value= 0.594), PPD (with P-value= 0.134), and microbial levels (P-value = 0.188) in both groups from the baseline.” However, none of the showed p-values mean statistical significance. The sentence is not understandable. Does the comparison mean within or between comparisons?

Response: we have now revised the result section and removed the following as they were baseline comparisons: that “statistically significant difference was noted in the Pi (P-value = 0.791), Gi (P-value= 0.594), PPD (with P-value= 0.134), and microbial levels (P-value = 0.188) in both groups.” Instead, we have now added the follow-up comparisons. The p-values are for intergroup comparison. The changes in the manuscript have been highlighted in track change mode in the revised manuscript.

  

- Conclusion may also be misleading. L. barbarum mouthwash presented similar clinical efficacy, when compared to chlorhexidine. The SRP performed may be the main reason to the so-called “effective in managing periodontal disease”. Please provide a interpretation aligned with your aims.
- Response: We thank the reviewers for their valuable suggestions. We have now revised the conclusion as follows: Goji berry mouthwash along with SRP was effective in reducing the Gi, Pi, BOP, and PPD. Goji berry mouth also increased the antioxidant levels in saliva in patients with periodontitis. Thus, goji berry mouthwash is a viable adjunct to SRP for patients with periodontitis. The changes in the manuscript have been highlighted in track change mode in the revised manuscript.

Introduction

- The term “exaggerate” in the first paragraph should be revised, as may be not be the most appropriate term in an epidemiological point of view. In addition, authors have cited both risk factors and indicator factors associated with periodontitis.

Response: We thank the reviewers for their valuable suggestions. We have now revised the term as follows: Apart from the microbial etiology, other risk factors that influence the severity of periodontal inflammation include smoking, diabetes mellitus, HIV, nutritional deficiency, medications, poor oral hygiene, and genetics. The changes in the manuscript have been highlighted in track change mode in the revised manuscript.

  

- Reference is necessary for the following sentence: “However, mechanical debridement is technically demanding, as many times removal of hard and soft tissue deposits from pockets greater than five mm becomes challenging.” In well-trained clinicians, this may not be a problem.
- Response: We have now added two references for this sentence as requested. The changes in the manuscript have been highlighted in track change mode in the revised manuscript.

Heitz-Mayfield L.J., Trombelli L., Heitz F., Needleman I., Moles D. A systematic review of the effect of surgical debridement vs. non-surgical debridement for the treatment of chronic periodontitis. J. Clin. Periodontol. 2002;29((Suppl. S3)):92–102. doi: 10.1034/j.1600-051X.29.s3.5.x.

 Sherman P.R., Hutchens L.H., Jr., Jewson L.G., Moriarty J.M., Greco G.W., McFall W.T., Jr. The effectiveness of subgingival scaling and root planing. I. Clinical detection of residual calculus. J. Periodontal. 1990;61:3–8. doi: 10.1902/jop.1990.61.1.3.

  

- Provide citation for the following sentences: “Recently, goji berry, commonly known as Wolfberry, Himalayan goji, or Tibetan goji, has gained a lot of popularity due to its strong antioxidant and anti-inflammatory properties.” And “The stems and berries of goji berry plant was effective against many various Gram-negative bacteria and Gram-positive bacteria.”
- Response: We thank the reviewer for their suggestion, We have now added reference no 38-48 , which discusses the antimicrobial and antioxidant properties of the goji berry as suggested. The changes in the manuscript have been highlighted in track change mode in the revised manuscript.

 

- Italicize the word “in vitro.”

Response: We thank you for your suggestion, We have now Italicized the word as requested.

  

- Reference #47 may not be necessary in the following sentence: “(…) efficacy of L. barbarum mouthwash along with SRP for patients with chronic periodontitis compared to chlorhexidine for the first time.47”

Response: We have removed reference no 47 from the sentences mentioned above.- 

  

- Why salivary levels of protein thiol were assessed in the present study? What is the relevance of the present study? This is not properly explained in this section.

Response: we thank the reviewers for their valuable insight and key appraisal. we have now added the importance of assessment of local antioxidant levels in oral cavity as immune defense mechanisms in the discussion and introduction section. Since protein thiol is an important antioxidant during periodontal disease and the ability of goji berry to increase it levels in saliva as additional advantage over chlorhexidine. The changes in the manuscript have been highlighted in track change mode in the revised manuscript.

  

- Objective: Pi, Gi, BOP, PPD, and CAL must be explained, as this is the first time they appear in the text.

Response: We have now added the full forms for each of the abbreviations in the objective section as suggested.

  

- Methods: Figure 1 does not represent the flowchart of the study, and does not follow what CONSORT recommends. Figure 1 is illustrative and important, but the sentence must be revised.

Response: We thank the reviewers for the recommendation, we have now revised the figure 1.

  

- Figure 2, in fact, must be placed in the Results section, as recommended by CONSORT.

Response: We thank the reviewer for their suggestion, we have now placed figure 2 in the result section.

  

- Do not use Stage 1, use “stage I.” Provide reference for this inclusion criterion

Response: We have now revised the term Stage 1 to Stage I as suggested.

  

- Do not mistake exclusion criteria as the opposite of inclusion criteria. For instance, if only generalized periodontitis was included. It is not necessary to exclude those with localized periodontitis.

Response: We thank the reviewer for their valuable insight. We have now revised the criteria as suggested : Participants in age group of 20-50 years diagnosed with stage I to stage II localized/generalized periodontitis (Grades A to B).

  

- What is “chlorhexidineg”?

Response: We apologise for the Typo error, we have now corrected it as Chlorhexidine

  

- Revise the following sentence: “Participants currently using anti-inflammatory, antibiotics, or analgesics in the last six months.” It seems that patients that used antibiotics in the last six months were included.

Response: We have now revised the sentence as follows: Participants who were on any anti-inflammatory, antibiotics, or analgesics in last six months were excluded

  

- Revise exclusion criteria #10, as the sentence is in the future.

Response: We have now revised the sentence as follows: Participants using any other oral hygiene agents (mouthwash or gels) were excluded to remove confounding bias arising due to the difference in plaque control measures

  

- Sample size calculation is not properly described. Readers to not know the necessary number of patients to detect important measures in the primary outcome. For instance, the effect size of 0.7 is referring to what outcome?

Response: We thank the reviewer for pointing our mistake and not mentioning the effect size linked outcome. The effect size was calculated based on clinical attachment loss.

  

- Why allocation stated uneven (30:27) at baseline (Figure 2), as a 1:1 allocation ratio was established in the Methods?

Response: We thank the reviewer for their insight. We apologize for the mistake in numbers mentioned in the concealment. Allocation was done at ratio of 1:1 as mentioned in the method. We have now corrected and uploaded the revised figure as suggested.

  

- In fact, allocation concealment is not properly explained.

Response: We thank the reviewer for their insight. We have now added an explanation on how the allocation concealment was done using opaque amber-colored bottles to mask the color of the mouthwash.

  

- Both types of mouthwash may be very distinct in general characteristics, such as taste, color, and visual aspects. Therefore, this referee does not understand the following sentence: “The participants were also blinded about their group.”

Response: We agree with the reviewer that both goji berry and chlorhexidine mouthwash, mouthwash were stored in similar amber-colored bottles to mask the color of the mouthwash. However, differences in the taste will remain as a major factor in patient blinding/ concealment

  

- It is well-established that the best periodontal examination must be performed in six sites per tooth. It is not clear why authors have performed it only in four.

Response: We agree with the reviewers and apologize for the ambiguity in the sentence we have performed periodontal examination on buccal, lingual, mesial, and distal (in the interdental area the probing was done both from the buccal and lingual side of the interdental area. We have now revised the sentence in the manuscript.

  

- Further information regarding chlorhexidine is necessary. Was it manufactured by the authors? What percentage was used? Provide details.

Response: We have added the percentage of chlorhexidine (0.2%)

  

- Chlorhexidine, such as those commercially available, must not be diluted in water. The lower efficacy of chlorhexidine must not be ruled out in the present study.

Response: We agree with the reviewers. however, the chlorhexidine used our study 0.2% was prepared and was diluted in 10 ml of water. 

  

- It is not clear for how long (who many seconds) volunteers should rinse.

Response: We have now added the time the mouthwash should be used in manuscript.

  

- Calibration, if any, is not reported.

Response: Since single trained investigator examined the baseline and follow-up visits, calibration was not done.

 Results

- In Table 3, why p-values for the comparison between groups are reported only for baseline?

Response: The p-values for comparison between groups at follow-ups and mentioned in the last columns of the same table.

  

- The following sentence should be better placed in the Methods: “(…) repeated measures of ANOVA with Greenhouse Geisser correction’ followed by a ‘post-hoc analysis with Bonferroni adjustment’.”

Response: We have now revised the statement and mentioned it in Methods section.

  

- Figure 3 and Table 3 are repetitive results. Both of them are not necessary.
- Response: We agree with reviewer suggestions. We have now removed the figure 3.

 

- Authors wrote: “One patient reported bitter taste of the mouthwash and discontinued the mouthwash.” What mouthwash?

Response: we have now added which group (Test group) patient reported bitterness.

  

- Discussion: Effectiveness was not tested. Do not mistake it with efficacy.
- Response: We have modified the title according to the suggestion and instead of effectiveness mentioned it as efficacy

 

- Only mild periodontitis patients were included in the present study. Do not generalize it. This type of patient is not discussed.
- Response: We have added this in the limitations this points our results

 

- The following sentences are not correct: “Based on the results, it was noted that goji berry mouthwash can control the gingival inflammation, BOP, and plaque formation at 15 days following SRP with statistically significant difference compared to chlorhexidine group. A significant in the Gi, Pi, BOP, PPD was noted in participants using goji berry mouthwash.” No differences between groups were detected.
- Response: We agree with the reviewers, we apologize for the error and interpreatation. we have now corrected the results section accordingly.

 

- Again, chlorhexidine is not largely used for the management of periodontitis. In fact, its use as adjuvant to periodontitis treatment is not well-established, and the previously mentioned clinical practice guideline do not recommend it.

Response: we thank the reviewers for their recommendation, we have now added these points to our introduction and discussion sections

  

- A broader discussion regarding the L. barbarum on periodontal tissues is warranted.

Response: We thank the reviewers for their recommendation. We have now added the role of the L. barbarum on periodontal pathogens, periodontal ligament fibroblast, neutrophils and wound healing

 References:

  Gan L, Zhang SH, Liu Q, et al.: A polysaccharide-protein complex from Lycium barbarum upregulates cytokine expression in human peripheral blood mononuclear cells. Eur. J. Pharmacol. 2003;471(3):217–222. 12826241 10.1016/s0014-2999(03)01827-2

  Bo R, Liu Z, Zhang J, et al.: Mechanism of Lycium barbarum polysaccharides liposomes on activating murine dendritic cells. Carbohydr. Polym. 2019;205:540–549. 30446138 10.1016/j.carbpol.2018.10.057

  Du X, Wang J, Niu X, Smith D, Wu D, Meydani SN. Dietary wolfberry supplementation enhances the protective effect of flu vaccine against influenza challenges in aged mice. J Nutr 2014;144: 224–9.35.

 Ren Z, Na L, Xu Y, Rozati M, Wang J, Xu J, et al. Dietary supplementation with lacto-wolfberry enhances the immune response and reduces pathogenesis to influenza infection in mice. J Nutr 2012;142:1596–602.

 There is no discussion regarding the very short follow-up period.

 Response: We agree with the reviewer and we have added these points as the limitations of the study

# Reviewer report: Reviewer response for version 1

**DOI:** 10.5256/f1000research.142608.r173857

**Published:** 2023-06-06

**Authors:** Rajinder K. Sharma, Deepti Anand

**Affiliations:** 1 Department of Periodontics and Oral Implantology, Postgraduate Institute of Dental Sciences, Rohtak, Haryana, India

**Abstract:******

 In abstract, results have been mentioned as significant in both the groups, however the p values mentioned don't corroborate the significant results. Please do the necessary corrections.

**Introduction:**

 It needs to be brief & relevant. The objectives of the study may be omitted in the introduction.

**Methodology**

 Please describe in detail the method of randomization of 57 participants in test group(30) and control group(27).

 Were the participants recruited on consecutive order or in pooled manner?

 Out of 30 participants in test group, 15 were dropped out, only 15 were included in statistical analysis. Sample size calculation was 27 patients in each group. Rationale of sample size calculation is not served in this study. 'Around' 60 participants were recruited; Please avoid vague terms like 'around'. Please mention 57.

 Out of 27 allocated to control group, 16 were lost to follow up hence only 11 are left for follow up completion, however in the flow chart it is mentioned, 14 participants were analyzed. Please look into it.

**Results**

 In the result section, demographic data is with reference to patients recruited whereas all other data is with reference to patients who completed the study. Similar data may be included in the result section, either all of the patients recruited or only the patients who have completed the follow up.

 All the results have been presented in overall average values. PPD is an important parameter influencing the outcomes of periodontal intervention. Data needs to be segregated as per PPD.

**Discussion:**

 Please focus the discussion on the results of the overall study period of one month, unless there is some interesting finding otherwise.

 It takes 6-8 weeks for healing to complete after SRP, Results of SRP are required to be estimated after this period. However the follow up of study period was one month; Please comment on it.

 A significant part of discussion is only review of literature with reference to Guji berry; Please avoid it as much as possible. Comment on the findings of your study may be the focus of discussion .

**Conclusion:**

 Conclusions are not based on the results of the study. For example "L.barbarum is a promising superfood with many potential health benefits", is not the conclusion of the study.

 L. barbarum has been investigated as an adjunct to SRP. Negative control is missing in the study. Hence the results can't be attributed exclusively to L.barbarum.

Is the work clearly and accurately presented and does it cite the current literature?

Yes

If applicable, is the statistical analysis and its interpretation appropriate?

Partly

Are all the source data underlying the results available to ensure full reproducibility?

Partly

Is the study design appropriate and is the work technically sound?

Yes

Are the conclusions drawn adequately supported by the results?

Partly

Are sufficient details of methods and analysis provided to allow replication by others?

Yes

Reviewer Expertise:

Periodontitis, periodontal medicine

We confirm that we have read this submission and believe that we have an appropriate level of expertise to confirm that it is of an acceptable scientific standard, however we have significant reservations, as outlined above.

# Response

**Published:** 2023-11-06

**Authors:** Aditi Chopra

**Affiliations:** Periodontology, Manipal College of Dental Sciences, Manipal, Manipal, India

Firstly, we would like to thank all the reviewers and the editor for spending their valuable time and providing us with their valuable comments and suggestions. We truly appreciate your guidance in improving our work. We have tried to address the comments and revised the manuscript accordingly. The changes in the manuscript have been highlighted in track change mode in the revised manuscript.

 Reviewer 1

- Abstract: In the abstract, results have been mentioned as significant in both the groups, however the p values mentioned don't corroborate the significant results. Please do the necessary corrections.

Response: We thank the reviewers for their valuable insight. We have now revised the abstract and interpretation of the results.

  

- Introduction: It needs to be brief & relevant. The objectives of the study may be omitted in the introduction.

Response: We thank the reviewer for their suggestion. We have now revised and shortened the introduction/background section in the abstract.

  

- Methodology: Please describe in detail the method of randomization of 57 participants in test group(30) and control group(27). Were the participants recruited on consecutive order or in pooled manner?

Response: We have now added the method of randomization (computer-generated random sequence) in the abstract. The participants were recruited in as when they come to the OPD and randomized once the consequent order.

  

- Out of 30 participants in test group, 15 were dropped out, only 15 were included in the statistical analysis. The sample size calculation was 27 patients in each group. The rationale of sample size calculation is not served in this study. 'Around' 60 participants were recruited; Please avoid vague terms like 'around'. Please mention 57.

Response: We thank the reviewer for their valuable suggestion. We have now revised paper accordingly and removed the word around along number of participants

  

- Out of 27 allocated to control group, 16 were lost to follow up hence only 11 are left for follow up completion, however in the flow chart it is mentioned, 14 participants were analyzed. Please look into it.

Response: We thank the reviewer for their valuable suggestion. We have now revised paper accordingly and provided the corrected CONSORT flow diagram

 Results

- In the result section, demographic data is with reference to patients recruited whereas all other data is with reference to patients who completed the study. Similar data may be included in the result section, either all of the patients recruited or only the patients who have completed the follow up.

Response: We thank the reviewer for their valuable suggestion. We have now revised paper accordingly and included the data related to the patients who were recruited

  

- All the results have been presented in overall average values. PPD is an important parameter influencing the outcomes of periodontal intervention. Data needs to be segregated as per PPD.

Response: We thank the reviewers for their valuable insight. However, since it study was conducted on a small number of participant where segregation of PPD was not possible due to similarity in the amount of PPD, we have mentioned the data as combine reduction in PPD from baseline, moreover with segregation we found that all the patient fall under 1-3 mm of PPD and CAL loss due to recession of 1-2 mm .

 Discussion:

- Please focus the discussion on the results of the overall study period of one month, unless there is some interesting finding otherwise. It takes 6-8 weeks for healing to complete after SRP, Results of SRP are required to be estimated after this period. However, the follow-up of study period was one month; Please comment on it.

Response: We thank the reviewer for the critical appraisal of these key findings. We have now added these points to the limitations of the study. We have kept the follow-up at 1 month as long-term use of chlorhexidine mouthwash is not recommended owing to the development of various side effects

  

- A significant part of discussion is only review of literature with reference to Goji berry; Please avoid it as much as possible. Comment on the findings of your study may be the focus of discussion.

Response: We thank the reviewers for their valuable insight. We have now revised the manuscript accordingly. We have shortened the evidence on goji berry and discussed the clinical implications of our results and limitations of the study

 Conclusion:

- Conclusions are not based on the results of the study. For example "L.barbarum is a promising superfood with many potential health benefits", is not the conclusion of the study.

Response: We thank the reviewers for their valuable insight. We have revised the conlucsion and removed the line suggested above.

  

- L. barbarum has been investigated as an adjunct to SRP. Negative control is missing in the study. Hence the results can't be attributed exclusively to L.barbarum.

Response: We thank the reviewers for their valuable insight and agree with fact that the results are due to the combined effects of SRP with goji berry mouthwash. Since SRP is primary and the fundamental step that should be performed and matched in both the group we aimed to compared goji berry mouthwash with chlorhexidine. We have added these points to our discussion as you have suggested.
